# Immunomodulatory effect of PLGA-encapsulated mesenchymal stem cells-derived exosomes for the treatment of allergic rhinitis

**DOI:** 10.3389/fimmu.2024.1429442

**Published:** 2024-07-08

**Authors:** Khawar Ali Shahzad, Zhao Wang, Xuran Li, Jiaojiao Li, Maoxiang Xu, Fei Tan

**Affiliations:** ^1^ Department of ORL-HNS, Shanghai Fourth People’s Hospital, School of Medicine, Tongji University, Shanghai, China; ^2^ Plasma Medicine and Surgical Implants Center, School of Medicine, Tongji University, Shanghai, China; ^3^ The Royal College of Surgeons in Ireland, Dublin, Ireland; ^4^ The Royal College of Surgeons of England, London, United Kingdom

**Keywords:** exosomes, mesenchymal stem cell, allergic rhinitis, PLGA, sustained release, immunomodulatory effect, peroxisome proliferator-activated receptor pathway

## Abstract

**Introduction:**

Allergic rhinitis (AR) is an upper airway inflammatory disease of the nasal mucosa. Conventional treatments such as symptomatic pharmacotherapy and allergen-specific immunotherapy have considerable limitations and drawbacks. As an emerging therapy with regenerative potential and immunomodulatory effect, mesenchymal stem cell-derived exosomes (MSC-Exos) have recently been trialed for the treatment of various inflammatory and autoimmune diseases.

**Methods:**

In order to achieve sustained and protected release of MSC-Exos for intranasal administration, we fabricated Poly(lactic-co-glycolic acid) (PLGA) micro and nanoparticles-encapsulated MSC-Exos (PLGA-Exos) using mechanical double emulsion for local treatment of AR. Preclinical *in vivo* imaging, ELISA, qPCR, flow cytometry, immunohistochemical staining, and multiomics sequencing were used for phenotypic and mechanistic evaluation of the therapeutic effect of PLGA-Exos *in vitro* and *in vivo*.

**Results:**

The results showed that our PLGA platform could efficiently encapsulate and release the exosomes in a sustained manner. At protein level, PLGA-Exos treatment upregulated IL-2, IL-10 and IFN-γ, and downregulated IL-4, IL-17 and antigen-specific IgE in ovalbumin (OVA)-induced AR mice. At cellular level, exosomes treatment reduced Th2 cells, increased Tregs, and reestablished Th1/Th2 balance. At tissue level, PLGA-Exos significantly attenuated the infiltration of immune cells (e.g., eosinophils and goblet cells) in nasal mucosa. Finally, multiomics analysis discovered several signaling cascades, e.g., peroxisome proliferator-activated receptor (PPAR) pathway and glycolysis pathway, that might mechanistically support the immunomodulatory effect of PLGA-Exos.

**Discussion:**

For the first time, we present a biomaterial-facilitated local delivery system for stem cell-derived exosomes as a novel and promising strategy for AR treatment.

## 1 Introduction

Allergic rhinitis (AR), characterized by nasal symptoms (e.g., sneezing, itching, nasal blockage, and rhinorrhea) and non-nasal symptoms, is an inflammatory upper airway disease caused by exposure to allergens (1, 2). AR usually co-occurs with asthma and/or conjunctivitis, and is a global health concern causing major social burden and economic impact. The hallmark of AR is an immunoglobulin E (IgE)-mediated, type I hypersensitivity reaction which triggers a cascade of immunological and biochemical events leading to clinical onset of the disease. Despite an abundance of research, there is no optimal regimen to cure AR, and most AR symptoms can only be managed to a certain level (3, 4).

Patient education, allergen avoidance, pharmacotherapy and allergen-specific immunotherapy (AIT) are all part of the conventional treatment for AR. Intranasal corticosteroids are first-line pharmacotherapy, whereas oral and/or topical H1-antihistamines and leukotriene receptor antagonists remain the second-line, all of which aim for relief of symptoms. AIT is the only treatment able to modify the natural course of AR through modulation of a complex interaction between the innate and adaptive immunity responses. However, for a sustained effect, AIT should be used for a minimum of 3 years, often leading to poor patient compliance and therapeutic outcomes (5).

Exosomes, especially those derived from mesenchymal stem cells (MSCs), have attracted the attention of numerous researchers and clinicians due to their unique ability to repair damaged tissue, attenuate inflammation, inhibit apoptosis, and module the immune system (6, 7). Exosomes are nanoscale, spherical, and lipid bi-layered single membrane extracellular vesicles, which act as intercellular messengers (8). Exosomes contain numerous functional cargoes, including proteins, glycoconjugates, lipids, nucleic acids, metabolites, and other bioactive substances. An exceptional advantage of exosome therapy is that exosomes can be biochemically modified to broaden, change, or improve their therapeutic effects.

Many preclinical studies and clinical trials have applied MSC-derived exosomes (MSC-Exos) for the treatment of various disorders. These include, but are not limited to, respiratory diseases (e.g., COVID-19 and acute respiratory distress syndrome) (9), musculoskeletal diseases (e.g., fracture and osteoarthritis) (10), nervous system diseases (e.g., ischemic stroke and spinal cord injury) (11, 12), skin diseases (e.g., wound healing and atopic dermatitis) (13), and even cancer (14). However, evidence of stem cell-derived exosome therapy on allergic airway conditions, such as AR and asthma, are very scarce. Few groups evaluated the therapeutic effect of human MSC-derived exosomes in inhibiting the Th2 cell differentiation via regulating the miRNA pathway in either AR or asthma (15, 16). These studies focused on Th2 cells only, whereas both Th1/Th2 imbalance and downregulation of regulatory T cells (Tregs) have more important and dynamic value during AR development.

MSC-derived exosomes could be administered either systemically or locally for disease treatment, each of these two modalities has advantages and disadvantages. Local delivery of exosomes might be more feasible for anatomical targets that are open to external environment either naturally or by surgery. A typical scenario would be transnasal approach for AR treatment. In addition, intranasal application of MSC-Exos could bypass vital organs, e.g., liver and kidney, while providing higher therapeutic concentration at nasal mucosa. However, even locally delivered exosomes might be easily degraded and rendered inactive, especially when the recipient environment is harsh, such as frequent sinonasal mucociliary clearance.

Therefore, biomaterial-assisted local and sustained release might be an ideal solution for exosome therapy of AR. Generally, the biomaterials available for exosome delivery are characterized according to their composition (e.g., hyaluronic acid, gelatin methacrylate), morphology (e.g., microneedle, nanoparticle), and stimuli-response (e.g., pH, temperature, protein), thereby allowing disease-specific customization (17). Poly(lactic-co-glycolic acid) (PLGA) has been proven as an excellent carrier of drugs and proteins for the treatment of various diseases because of its outstanding biodegradability and biocompatibility (18–20).

From a mechanistic perspective, it has been proven that treatment with MSC-Exos could increase the levels of anti-inflammatory cytokines (e.g., IL-10 and TGF-β) and decreased that of Th2 cytokines (e.g., IL-4) and pro-inflammatory cytokines (e.g., IL-17) in inflammatory bowel disease (21). Furthermore, MSC-Exos treatment could elevate the percentage of CD4^+^CD25^+^Foxp3^+^ Tregs in acute colitis (22). It is therefore speculated that MSC-Exos possess immunomodulatory capacity manifested by various immune cellular responses also during management of AR.

For the first time, we developed a local delivery platform by fabricating different sizes of PLGA-encapsulated exosomes (PLGA-Exos) micro and sub-micron particles using the mechanical double emulsion method. PLGA-Exos were then administered intranasally to evaluate their sustained release and therapeutic capabilities for AR *in vitro* and *in vivo*. In addition, multiomics sequencing was performed for mechanistic interpretation. Through multilevel evaluation (i.e., tissue, cell, protein, and gene levels), we present our biomaterial-facilitated local delivery system for stem cell-derived exosomes as a novel and promising strategy for immunomodulatory AR treatment.

## 2 Materials and methods

### 2.1 Cell lines, animals, and reagents

Six to eight weeks old male Balb/c mice were purchased from Vital River Animal Technology Co., Ltd (Beijing, China) and housed in specific pathogen free (SPF) Laboratory Animal House of Tongji University (Shanghai, China). All animal experiments and procedures were conducted following the ethical guidelines of the School of Medicine, Tongji University (Permit no. T3-HB-LAL-2023-23) and recommendations of Guide for the Care and Use of Laboratory Animals of Ministry of Science and Technology of the People’s Republic of China.

Human nasal epithelial cells (HNEpCs) were purchased from Procell Life Science and Technology Co., Ltd (Wuhan, China) (Cat NO.: CP-H252) and THP-1 cell line was obtained from Chinese Academy of Sciences Typical Culture Preservation Committee Cell bank (ATCC, Shanghai, China) and maintained in MEM (Shanghai BasalMedia Technologies, Shanghai, China) supplemented with 10% fetal bovine serum (FBS) (Gibco, Shanghai, China) and 1% penicillin-streptomycin (Shanghai BasalMedia Technologies, Shanghai, China) in humidified conditions with 5% CO_2_ and 95% air at 37 °C. The RPMI2650 cell line was provided by Jennio Biotech Co., Ltd (Guangzhou, China) and grown in RPMI1640 with L-glutamine (Corning, Manassas, USA) supplemented with 10% FBS. Human umbilical cord derived mesenchymal stem cells (HUC-MSCs) were purchased from Cytoniche Biotechnology Co., Ltd (Beijing, China) and maintained in Dulbecco’s modified eagle medium (DMEM) (Biosharp, Beijing, China) supplemented with 10% FBS.

### 2.2 Isolation and identification of MSC-derived exosomes

HUC-MSCs-derived exosomes (MSC-Exos) were isolated from the collected culture medium by ultracentrifugation as described previously (23, 24). Briefly, HUC-MSCs were cultured with DMEM containing exosome-free FBS (A27208-03, Gibco, Shanghai, China). When the cell confluency reached 80-90%, the culture medium was collected after 48 hr and centrifuged at 10,000 g for 30 min to remove the dead cells and cell debris. The culture medium was then filtered through 0.22 µm pore sized sterile filters (Millipore, Billerica, MA, USA) to ensure the depletion of large particles. The filtered culture medium was then centrifuged at 100,000 g for 90 min to obtain the pellet of exosomes. The pellet was resuspended in sterile PBS, centrifuged again at 100,000 g for 90 min, and stored at -80 °C until future use.

The morphology and size distribution of isolated MSCs-Exos were analyzed using transmission electron microscopy (TEM) (FEI F20, Columbus, USA) and nanoparticle tracking analysis (NTA) (NS300, NanoSight, Malvern, UK), respectively (24). Western blotting was performed to determine the expression of CD81, CD63, HSP70 and calnexin (Proteintech, USA) protein markers in the extracted MSCs-Exos (25).

### 2.3 Preparation and characterization of blank, ICG-encapsulated, and exosome-encapsulated PLGA micro/sub-micron particles

PLGA particles of different sizes (1 µm, 800 nm, 400 nm and 200 nm) with or without encapsulation of indocyanine green (ICG) (Macklin, Shanghai, China) or exosomes were prepared using double emulsion method as described previously (26, 27) with minor modifications. In brief, 100 mg of 0.67 dL/g carboxy-terminated 50:50 PLGA polymer (Daigang Co, Jinan, China) was dissolved completely in dichloromethane (Titan Scientific, Shanghai, China), and 50 µL ICG solution (20 mg/mL, in DMSO) or MSCs-Exos (3 µg, 5 µg, 10 µg or 50 µg/mg PLGA) were added and sonicated using an ultrasonic homogenizer (JY92-IIN, Ningbo Xinzhi Biotech., Ningbo, China) at 90% for 6 min (10 sec sonication and 15 sec break). The mixture was then added in 50 mL 1% poly vinyl alcohol (PVA) (Macklin, Shanghai, China) and sonicated again at 90% for 6 min in case of 200 nm, 4 min for 400 nm, 2 min for 800 nm, and 1.5 min for 1µm sized PLGA particles (10 sec sonication and 15 sec break). Finally, 100 mL dd H_2_O was added in the solution and placed overnight on a magnetic stir bar to evaporate the dichloromethane. Finally, the solution was centrifuged (at 3000 rpm for 1 µm, 6000 rpm for 800 nm, 9000 rpm for 400 nm, and 12000 rpm for 200 nm particles) to collect the PLGA micro/sub-micron particles (PLGA-MPs/SMPs). All sizes of particles with ICG encapsulated PLGA-MPs/SMPs (ICG-PLGA MPs/SMPs) were centrifuged at 20,000 rpm for 10 min to remove the unencapsulated ICG. The resulting PLGA particles were stored at -20 °C. ICG-labelled exosomes (ICG-Exos) for *in vivo* tracking were prepared by incubating 10 µg/mL ICG in 50 µg exosomes for 12 hr at 4 °C (28, 29). The exosomes were centrifuged at 100,000 g for 90 min by adding sterile PBS to remove the unattached ICG.

Scanning electron microscopy (SEM) (Hitachi, S-4800, Japan) was performed to visualize the surface morphology and shape of prepared PLGA-MPs/SMPs. The size distribution and zeta potential were analyzed using dynamic light scattering (DLS) (OPT2301140, Opptronix, Shanghai, China).

### 2.4 *In vivo* sinonasal retention of PLGA-MPs/SMPs and *in vitro* exosome release

In order to evaluate the sinonasal retention of ICG-Exos and different sized (1 µm, 800 nm, 400 nm and 200 nm) ICG-PLGA MPs/SMPs, 50 µg of ICG-Exos or 5 mg of ICG-PLGA-MPs/SMPs were suspended in 30 µL PBS and administered intranasally (15 µL in each nasal cavity) into every Balb/c mouse. The mice were placed under anesthesia by isoflurane inhalation for *in vivo* imaging of whole head using InVivo Smart-LF system (VISQUE, B12BAA002, Korea) at different time points (30 min, 2 hr, 6 hr, 12 hr, 24 hr, 48 hr, 72 hr, 96 hr, 120 hr and 168 hr). The exposure time was 1 min, whereas excitation and emission wavelengths were 740-790 nm and 810-860 nm, respectively. Three mice for each group were imaged for statistical analysis.

The *in vitro* release profile of exosomes from 800 nm sized PLGA SMPs encapsulated exosomes (PLGA-Exos) with different concentrations of exosomes (3 µg, 5 µg, 10 µg or 50 µg/mg PLGA) over time was measured for 11 days. A BCA assay kit (Beyotime, Shanghai, China) was used to assess the release profile. Based on the values, a cumulative release curve was plotted (n=3). In brief, 5 mg of PLGA-Exos with different concentrations were added in sterile 1.5 mL Eppendorf tube with 1 mL PBS and PLGA-Exos were suspended thoroughly. The tubes were placed in an incubator at 37 °C and 20 µL supernatant was replaced with fresh PBS by centrifugation at 12,000 g for 10 min on days 1, 2, 3, 4, 5, 6, 7, 8, 9, 10 and 11. The exosomes release was determined by the released protein content.

### 2.5 Cellular uptake of PLGA-Exos

RPMI2650 cell line was used as nasal epithelial cells, whereas THP-1 cells stimulated by phorbol-12-myristate-13-acetate (PMA) (Sigma-Aldrich, MO, USA) were used as macrophages in the *in vitro* models. 100 ng/mL of PMA was added in 1×10^6^ THP-1 cells for 24 hr in an incubator at 37 °C with 95% air and 5% CO_2_ to obtain macrophages. PLGA-Exos labelled with PHK26 fluorescent dye (Solarbio, Beijing, China) were co-cultured with RPMI2650 and THP-1 cells. Briefly, RPMI2650 and THP-1 cells were cultured in 15 mm polystyrene cell culture dish (NEST, USA) for 24 hr until 80-90% confluency was attained. The cells were co-cultured with 50 µL of 800 nm PLGA-Exos (5 mg PLGA, 10 µg exosomes/mg PLGA) after adding 500 µL culture medium for 4 hr, 12 hr, 24 hr and 48 hr. Likewise, THP-1 cells were also co-cultured with 50 µg PHK26 labelled exosomes. Cells were fixed with 4% paraformaldehyde (PFA) (Titan, Shanghai, China) for 30min at room temperature (RT), incubated with β-actin (Abcam, Cambridge, UK) primary antibody overnight at 4 °C, and stained with Alexa Fluor^®^ 488 (Abcam, Cambridge, UK) secondary antibody for 1 hr at RT. DAPI was used to stain the nuclei of the cells. Finally, the cells were imaged using a laser scanning confocal microscope (LSCM) (ECLIPSE Ti2, Nikon, Japan).

### 2.6 *In vitro* therapeutic effects of PLGA-Exos

HNEpCs were cultured in 12-well plate at a density of 1×10^5^ cells/well in 500 µL culture media. The culture media were removed completely after 12 hr of incubation when 90% confluency was obtained. Medium with 0.1 µg/mL lipopolysaccharide (LPS) was added in the wells of positive control and treatment group, whereas unstimulated cells were considered as negative control. After 24 hr of stimulation, PLGA-Exos (5 mg/well) were added in the treatment group and incubated for further 24 hr. The culture media was aseptically collected from wells of each group and centrifuged at 3000 rpm for 5 min to remove the cell debris and supernatant, and then preserved at -80 °C until assayed. Enzyme linked immunosorbent assay (ELISA) and real time quantitative PCR (RT-qPCR) was performed to analyze the effect of PLGA-Exos treatment at protein and transcriptomic levels, respectively.

### 2.7 Establishment of mouse AR model and treatment regime

The AR model was established as described previously (30, 31) with minor modifications. Briefly, Balb/c mice were randomly divided into 5 groups, i.e., negative control (NC, normal mice), positive control (OVA, AR mice), blank PLGA (PLGA), exosomes (Exos) and exosomes-encapsulated in PLGA sub-micron particles (PLGA-Exos) groups (n = 12/group). Mice from the last 4 groups were first subjected to basic sensitization by intraperitoneal injection of 200 µL of Grade V ovalbumin (OVA) and aluminum hydroxide (Al(OH)_3_) (Sigma-Aldrich, GmbH Germany) (1 mg/mL OVA+100 mg/mL Al(OH)_3_) on day 0, 7 and 14. These mice were then challenged for 7 consecutive days with intranasal administration of 20 µL OVA solution (40 mg/mL) (10 μL in each nostril) on a daily basis up to day 21. The control group (NC) was sensitized and challenged with the same amount of PBS at a same frequency.

On day 22 (day 0 for treatment), all 5 groups were intranasally administered with individual regimen (NC and OVA with PBS, PLGA with Blank PLGA, Exos with only exosomes and PLGA-Exos with PLGA encapsulated exosomes) with a total volume of 20 µL after every five days for six sessions. The AR symptoms and signs, i.e., nasal scratching, sneezing and number of eosinophils in nasal lavage fluid, of 6 mice from each group were observed, recorded and scored after the last treatment for a period of 20min by a blinded observer (**Supplementary Figure S1**). The nasal tissues, spleens, blood samples from each treatment group were collected at 2 (after 3 sessions of treatment) and 4 weeks (after 6 sessions of treatment).

### 2.8 Enzyme-linked immunosorbent assay

The concentrations of IFN-γ (Servicebio®, Wuhan, China), IL-2, IL-4, and IL-10 (EK102-01, EK104-03, EK110/2-02; Multi Sciences, Hangzhou, China) in the HNEpCs cell culture supernatant were measured using ELISA as per manufacturer’s protocol. Blood sample collected after eyeball removal from mice of each treatment group was placed for 3 hr at 4 °C and then centrifuged at 3000 rpm for 15 min to obtain serum. The isolated serum was stored at -80 °C until further use. The serum was analyzed for IFN-γ, IL-4, (GEM0006, GEM0006; Servicebio®, Wuhan, China), IL-10 (88-7105; Invitrogen, Vienna, Austria), IL-17 (EK217/2-01; Multi Sciences, Hangzhou, China) and sIgE (Konodi Bio, Xiamen, China) concentrations using ELISA according to given protocols.

### 2.9 RNA extraction and RT-qPCR analysis

Total RNA was isolated from the cultured cells and spleen tissues of each treatment group using an RNA-Quick Purification Kit (ES Science, Shanghai, China). The concentration of RNA isolated from each sample was quantified using a NanoDrop1000 Spectrometer (ThermoScientific, Waltham, MA, USA). 1 µg of RNA from each sample was used to synthesize cDNA by reverse transcription using a HiScript 1^st^ Strand cDNA Synthesis Kit (Vazyme, Nanjing, China). The RT-qPCR was performed using ChamQ Universal SYBR qPCR Master Mix (Vazyme, Nanjing, China). The primer sequences for HNEpCs IFN-γ, IL-2, IL-4, IL-10, and GAPDH and mice IFN-γ, IL-2, IL-4, IL-10, IL-17 and GAPDH are presented in **Supplementary Tables S1**, **S2**, respectively. The expression of mRNA was evaluated using RT-qPCR on an ABI 7500 Real-Time PCR System (Applied Biosystems, California, USA). The amplification conditions for PCR were 95 °C for 30 sec, followed by 40 cycles of denaturation at 95 °C for 10 sec and annealing at 60 °C for 20 sec. Housekeeping gene (GAPDH) was used to calculate the targeted gene expression levels and data was analyzed using the comparative ΔΔCT method.

### 2.10 Histopathological analysis of nasal tissues

Nasal tissues collected from mice of different treatment groups (2 and 4 weeks) were fixed in 4% PFA for 5 days at 4 °C and then decalcified by EDTA decalcification buffer (Cat#G1105, Servicebio, Wuhan, China) for 2-3 days at RT in a shaking incubator. The tissues were then dehydrated and embedded in paraffin, cut into sections of 4 µm thickness using a microtome, and observed under light microscope (Nikon) using routine H&E and PAS staining. The mean numbers of eosinophils and goblet cells were calculated by counting 10 different fields at 400× magnification, using Image-Pro Plus software (Media Cybernetics, Rockville, MD).

### 2.11 Flow cytometry

Spleens collected at 2 and 4 weeks from mice of different treatment groups (n=6) were processed to isolate the lymphocytes using a lymphocyte separation buffer (Cat#562574, BD Biosciences, San Diego, USA) as per manufacturer’s protocol. The isolated lymphocytes were stained by surface staining with anti-mouse APC-CD4 (Cat#116014, Biolegend), FITC-CD19 (Cat#152404, Biolegend) and PE/Cyanine7-CD25 (Cat#102016, Biolegend) for 30min at 4°C. After fixation for 45 min at 4 °C using a fixation buffer (Cat#420801, Biolegend), the intracellular staining was performed with anti-mouse PE-IFN-γ (Cat#505808, Biolegend), Brilliant Violet 605-IL-4 (Cat#504126, Biolegend) and Brilliant Violet 421-Foxp3 (Cat#126419, Biolegend) for 1 hr at 4 °C. Finally, flow cytometric analysis was performed using a LSRFortessa™ Cell Analyzer (Cat#647794L6, BD Biosciences, San Jose, USA) and data was analyzed using the v7.6.1 FlowJo software.

### 2.12 RNA sequencing for nasal tissues

The total RNA was extracted using a Trizol reagent kit (Invitrogen, USA). Extracted mRNA was enriched by Oligo(dT) beads fragmented into short fragments and reversely transcribed into cDNA using a NEB Next Ultra RNA Library Prep Kit for Illumina (New England Biolabs, USA). PCR and size selection by agarose gel electrophoresis were applied to construct the cDNA library. The library was sequenced using Illumina Novaseq6000 by Shanghai Wancheng Biotechnology Co., Ltd. The reads obtained from the sequencing machines were aligned to the reference genome using HISAT2. 2.4.

Principal component analysis (PCA) was performed using ‘gmodels’ package. DESeq2(v1.4.5) was used for differential expression analysis. The genes with false discovery rate (FDR) below 0.05 and fold change ≥ 2 was identified as differentially expressed genes (DEGs). The ‘ggplot2’ and ‘ImageGP’ were used to illustrate these DEGs when comparing different groups. Gene Ontology (GO), Kyoto Encyclopedia of Genes and Genomes (KEGG) analysis were performed by Phyper to uncover the underlying molecular mechanism.

### 2.13 Small RNA sequencing of exosomes

Small RNAs from MSC-Exos were obtained using a QIAzol kit (QIAGEN, Shanghai, China). Sequencing libraries were generated from each sample using RNeasy MinElute spin column. The small RNAs were sequenced using next-generation sequencing technology and the raw sequencing data were aligned with the database to identify and analyze the small RNA sequence. RNAhybrid, miRanda and TargetScan were used to accurately predict the target genes of miRNAs. The functional analyses were conducted as described in section 2.12.

### 2.14 Liquid chromatography-mass spectrometry analysis

SDT buffer (bioWORLD, Inc., USA) was used to extract protein from MSC-Exos. The protein was digested by trypsin according to the filter-aided sample preparation (FASP) procedure as described by Burnner et al., 2022 (32). Liquid chromatography-mass spectrometry (LC-MS) was performed on a Q Exactive mass spectrometer (Thermo Scientific, USA). The LC-MS data was acquired using a data-dependent method. The most abundant precursor ions from the survey scan (300–1800 m/z) were acquired for fragmentation and the data of LC-MS were identified using the MaxQuant 1.5.3.17 software. In addition, the sequences of protein were searched in the InterProScan software to identify the domain signatures.

Immune microenvironment analysis was performed using the R software. The core function utilized CIBERSORT deconvolution algorithm to dig immune cells from a gene expression matrix in samples. Stacked bar plots were used to illustrate the relative abundance of different immune cell subsets in the NC, OVA and PLGA-Exos groups.

### 2.15 Statistical analysis

The data were statistically analyzed, and figures were generated using GraphPad Prism 6.0 software (GraphPad, La Jolla, CA, USA). All the data were compared using one-way analysis of variance (ANOVA) and then by Tukey’s *post-hoc* test. All the data were expressed as mean ± SD. The *p-*value < 0.05 was considered statistically significant. All the experiments were repeated in triplicates unless otherwise mentioned.

## 3 Results

### 3.1 MSC-derived exosomes and different sized blank, ICG-PLGA MPs/SMPs, and PLGA-Exos MPs/SMPs are successfully collected and identified

**Figure 1:**
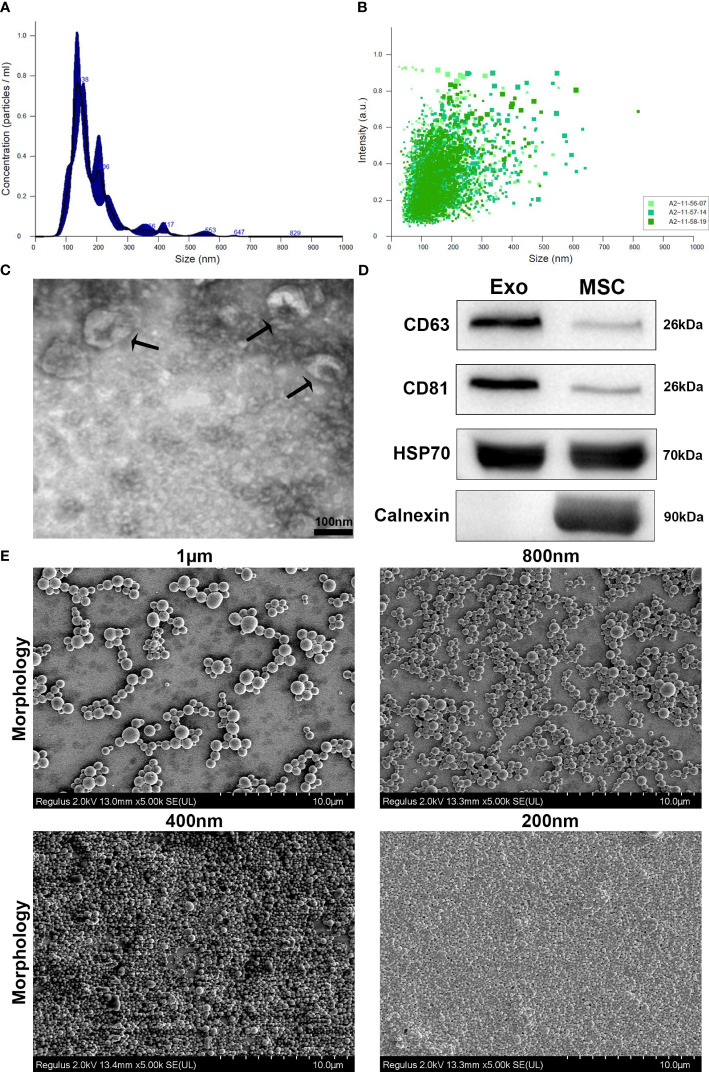
Characterization and phenotypic analysis of MSCs-Exos and different-sized PLGA MPs/SMPs. **(A, B)** Size distribution analysis of MSCs-Exos particles by nanoparticle tracking analysis (NTA); 103 ± 63nm was the mean size ± SEM for MSCs-Exos. **(C)** TEM image presenting shape of MSCs-Exos; scale bar 50 nm. **(D)** Surface markers detection of MSCs-Exos by western blot analysis. **(E)** Representative SEM micrographs for the morphology of different-sized PLGA MPs/SMPs; scale bar 10 µm. **(F, G)** Size and zeta potential distribution analysis of different-sized PLGA MPs/SMPs by DLS. (Exo, Exosomes; MSC, MSC cells) (n=3, performed in triplicate).

Exosomes derived from HUC-MSCs (MSCs-Exos) were identified using NTA, TEM and western blot analysis (10). Firstly, the size distribution was determined using NTA, which indicated that more than 90% of the exosomes were between 30 nm to 150 nm in diameter (**Figures 1A, B**). Secondly, as expected, a double layered membrane structure with cup-shaped morphology was revealed using TEM (**Figure 1C**). Finally, western blotting exhibited that the exosomes were positive for CD63, CD81 and HSP70 but negative for calnexin (**Figure 1D**).

All four different sizes of blank PLGA MPs/SMPs, ICG-PLGA MPs/SMPs, and PLGA-Exos MPs/SMPs fabricated using double emulsion method displayed smooth surface morphology and spherical shape (**Figure 1E**). The average sizes and zeta potentials of blank PLGA MPs/SMPs were 1013.5 nm, 797.3 nm, 400.1 nm and 198.1 nm, and -13 ± 5.81 mV, -12.2 ± 6.13 mV, -7.2 ± 7.32 mV and -4.1 ± 7.01 mV, respectively (**Supplementary Figures S2A, B**; **Table 1**). In comparison to blank ones, ICG-PLGA MPs/SMPs had similar sizes but slightly lower zeta potential (-15.6 ± 2.81 mV, -14.0 ± 5.32 mV, -10.1 ± 1.92 mV and -7.2 ± 2.40 mV, respectively) (**Supplementary Figures S3A, B**), while successfully presenting green fluorescence under fluorescence microscopy (data not provided). Moreover, the PLGA-Exos MPs/SMPs were comparatively bigger in size and lower in zeta potential when compared with blank ones (1073.9 nm, 852.7 nm, 480.8 nm and 250.1 nm and -19.8 ± 3.53 mV, -15.6 ± 1.35 mV, -14.2 ± 2.97 mV and -9.4 ± 3.21 mV, respectively) (**Supplementary Figures S4A, B**).

**Table 1:** Characterization of different-sized blank PLGA particles.

Sr. #	Average size of PLGA particle (nm)	Zeta potential (mV)
1	1013.5 ± 10.13	-13 ± 5.81
2	797.3 ± 12.50	-12.2 ± 6.13
3	400.1 ± 9.19	-7.2 ± 7.32
4	198. ± 11.92	-4.1 ± 7.01

### 3.2 PLGA-MPs/SMPs are capable to prolong intranasal retention *in vivo* and sustained release of exosomes *in vitro*

**Figure 2:**
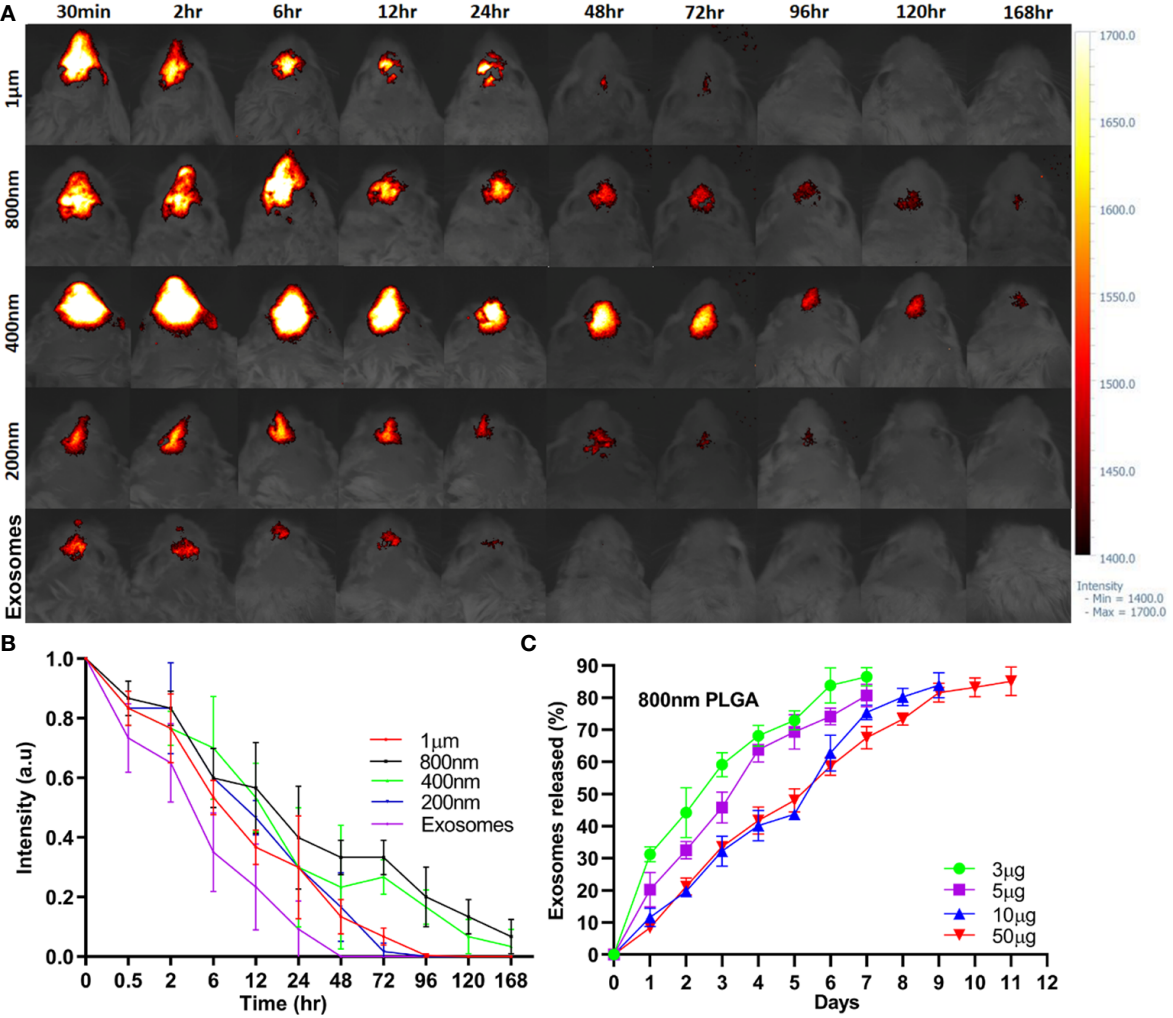
*In vivo* sinonasal retention of PLGA-MPs/SMPs and *in vitro* exosomes release. **(A)** ICG-encapsulated different-sized PLGA MPs/SMPs were administered intranasally followed by fluorescence imaging of the head in Balb/c mice at different time points. **(B)** The fluorescence intensity was measured to determine the retention time of ICG-PLGA MPs/SMPs. **(C)** *In vitro* release analysis of different concentrations of exosomes (3 µg, 5 µg, 10 µg or 50 µg/mg PLGA) from different-sized PLGA MPs/SMPs over time. All the data are presented as mean ± SD (n = 3).

The intranasal retention of ICG-Exos and different sized ICG-PLGA MPs/SMPs in Balb/c mice was evaluated using *in vivo* near-infrared imaging. As demonstrated in **Figures 2A, B**, strong fluorescence was observed in all sizes of ICG-PLGA MPs/SMPs up to 6 hr after intranasal administration. Compared with 1 µm and 200 nm sized ICG-PLGA MPs/SMPs, the ones in 800 nm and 400 nm sizes exhibited longer retention (120 hr vs. 48 hr). The quick disappearance of 1µm sized PLGA particles might be the larger size, the epithelial cells and macrophages cannot uptake MPs easily and can be cleared quickly from the nasal cavity by mucociliary clearance (33, 34), on the other hand, 200 nm sized PLGA particles were supposed to be uptaken by nasal epithelial cells and macrophages easily but degraded quickly because of very small diameter (35). Whereas, in case of ICG-Exos group strong fluorescence was observed up to 2 hr time point following complete disappearance after 24 hr.

*In vitro* release of exosomes from 800 nm PLGA-Exos SMPs with varying exosome concentrations (3 µg, 5 µg, 10 µg, and 50 µg/mg PLGA) was measured using the BCA quantification method (**Figure 2C**). *In vitro* exosomes release PLGA SMPs was completely concentration based as higher concentration (50 µg, and 10 µg) were released after 9 and 11 days respectively, whereas 5 µg, and 3 µg were released after 7 days.

Considering the above results in various aspects, i.e., *in vivo* retention time (800nm/400nm > 200 nm/1 µm), and *in vitro* exosome release (10 µg/50 µg > 3 µg/5 µg), PLGA-SMPs in 800 nm with 10 µg/mg exosomes concentration were determined to be most suitable for AR treatment and used thereafter.

### 3.3 The uptake of PLGA-Exos by nasal epithelial cells and macrophages peaked at 24hr

**Figure 3:**
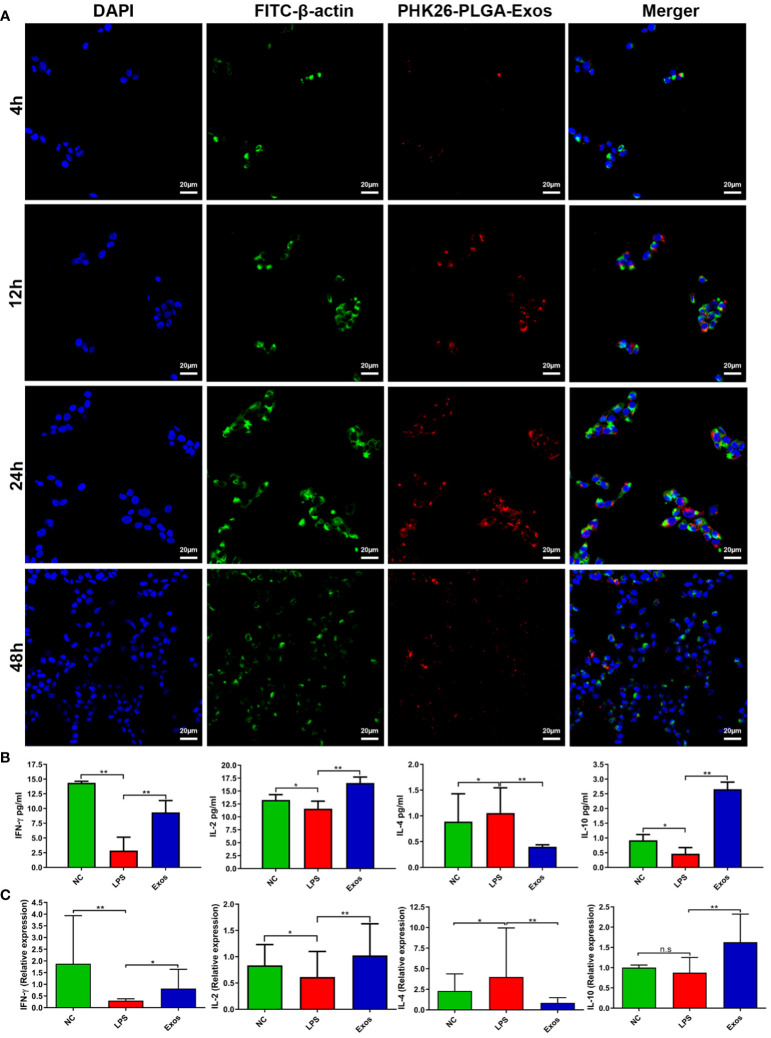
Cellular uptake and evaluation of *in vitro* therapeutic effect by PLGA-Exos. **(A)** Immunofluorescence images by confocal microscopy presenting the internalization of PLGA-Exos in THP-1 macrophages; scale bar 20 µm. **(B)** Quantification of IFN-γ, IL-2, IL-4, IL-10, and LPS levels in HNEpCs by ELISA. **(C)** Relative mRNA expression levels for IFN-γ, IL-2, IL-4, and IL-10 in HNEpCs by RT-qPCR. (NC, negative control; LPS, positive control; Exos, PLGA-Exos treatment group) (n=3, performed in triplicate) (*P < 0.05 and ** *P* < 0.01) (n.s, not significant).

PLGA-Exos labelled with PHK-26 fluorescent dye were co-cultured with RPMI2650 and THP-1 cells. Confocal imaging was performed to visualize the uptake of only exosomes and PLGA-Exos at 4, 12, 24 and 48 hr. The results demonstrated that PLGA-Exos were gradually internalized by RPMI2650 and THP-1 cells (**Figure 3A**; **Supplementary Figure S5**). It was observed that the uptake of PLGA-Exos was maximum at 24 hr followed by a decline of fluorescence at 48 hr in both nasal epithelial cells and macrophages (**Figure 3A**; **Supplementary Figure S5**). Whereas, maximum uptake of only exosomes was observed at 12 hr followed by a decline of fluorescence at 24 hr and 48 hr, respectively (**Supplementary Figure S6**). When dye-loaded PLGA particles were incubated with cells at 4 °C, where energy-dependent processes slow down, no intracellular fluorescence is expected. Any intracellular fluorescence obtained at 4 °C incubation would indicate the free dye that leaked out of the particles and diffused into the cells. This is an important issue (dye leakage) and is particularly relevant for *in vivo* biodistribution/clearance studies and needs to be addressed. In addition, our findings in uptake pattern are inconsistent with other studies (36, 37). Future work needed to be performed to clarify this issue.

### 3.4 PLGA-Exos treatment upregulates IFN-γ and IL-2 and IL-10 cytokines and downregulates IL-4 cytokine in a cellular model

LPS-stimulated HNEpCs were used as an *in vitro* model for the therapeutic effect of PLGA-Exos treatment. The Th1/Th2 balance was determined by the level of Th1-associated cytokines (e.g., IFN-γ and IL-2) and Th2- associated cytokines (e.g., IL-4). PLGA-Exos treatment increased the levels of IFN-γ, IL-2 and IL-10, and decreased those of IL-4 (**Figure 3B**). Moreover, the results of RT-qPCR also aligned with ELISA results (**Figure 3C**). These data suggested that PLGA-Exos treatment upregulated Th1 cytokines and downregulated Th2 cytokines in a cellular model.

### 3.5 PLGA-Exos treatment modulates Th1/Th2 cytokines balance, promotes IL-10 cytokines and decreases IL-17 cytokines and IgE of OVA-induced AR mice

**Figure 4:**
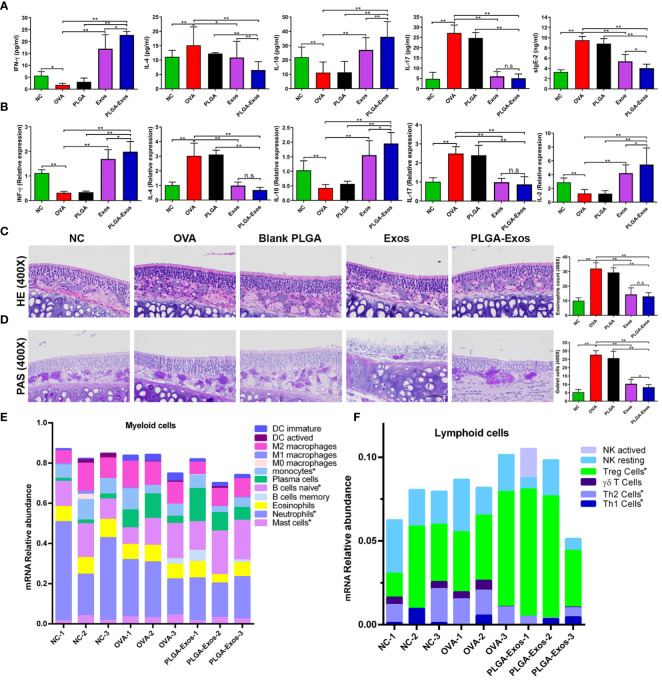
Effect of PLGA-Exos treatment on histopathological changes in nasal tissues, blood serum, and spleen cells in AR mice at 4 weeks’ time point. **(A)** Quantification of IFN-γ, IL-4, IL-10, IL-17, and sIgE in blood serum of AR mice by ELISA. **(B)** Relative mRNA expression levels for IFN-γ, IL-4, IL-10, IL-17, and IL-2 in spleen cells of AR mice. **(C, D)** Representative images of HE and PAS staining of nasal tissues representing the infiltration of inflammatory cells (eosinophils and goblet cells) in OVA group, which were reduced in treatment groups (Exos and PLGA-Exos); Magnification 400X. **(E)** Cibersort analysis of the mRNA expression relative abundance in myeloid cells. **(F)** Cibersort analysis of the of mRNA expression relative abundance in lymphoid cells. For a detailed comparison of gene expression of myeloid and lymphoid cells please see **Supplementary Figure S7**. (NC, negative control/normal mice; OVA, positive control/AR mice; Blank PLGA, AR mice treated with blank PLGA; Exos, AR mice treated with only exosomes; PLGA-Exos, AR mice treated with only exosomes-encapsulated in PLGA sub-micron particles) groups (n=6) (*P < 0.05 and ** *P* < 0.01) (n.s, Not Significant).

The allergic response in the blood serum of OVA-challenged AR mice is characterized by elevated Th2 cytokines (e.g., IL-4, IL-13) and antigen-specific IgE, and decreased Th1 cytokines (e.g., IFN-γ, IL-2) (38). In our study, the levels of IL-4, IL-17 and sIgE increased whereas those of IFN-γ and IL-10 decreased in the OVA group, suggesting a successful establishment of AR model. As shown in **Figure 4A** and **Supplementary Figure S7A**, treatment with Exos alone and PLGA-Exos significantly decreased the levels of IL-4, IL-17 and sIgE but increased those of IFN-γ and IL-10 at both 2- and 4-weeks. Additionally, a significant increase in the levels of IFN-γ and IL-10 and decrease in IL-4 and sIgE were observed in PLGA-Exos treatment group when compared with Exos alone group at 4-weeks.

Likewise, RT-qPCR was also performed at 2- and 4-weeks to detect the mRNA expression of IL-2, IL-4, IL-10, IL-17 and IFN-γ in the spleen of mice from different treatment groups. The RT-qPCR results were in accordance with the ELISA results as manifested by upregulated IL-2, IL-10, IFN-γ and downregulated IL-4 and IL-17 (**Figure 4B**; **Supplementary Figure S7B**). The reduction of IL-17 after PLGA-Exos treatment was more evident at 4 weeks (6 sessions of treatment) when compared to 2 weeks (3 sessions of treatment). After 6 treatments (4-weeks), significantly higher levels of mRNA expression in IFN-γ and IL-10 and lower IL-2 level were observed when PLGA-exos group was compared with Exos group.

### 3.6 PLGA-Exos treatment attenuates inflammatory cells infiltration in the nasal mucosa of AR mice

The main histopathological manifestation in nasal mucosa of AR is the accumulation and infiltration of various immune cells (39). The number of eosinophils and goblet cells increased in the OVA group but reduced in the Exos alone and PLGA-Exos treatment groups (**Figures 4C, D**; **Supplementary Figures S7C, D**). Moreover, a significant decrease in goblet cells was observed in PLGA-Exos group when compared with Exos only group. The results indicated that treatment with Exos and PLGA-Exos significantly inhibited the recruitment of inflammatory cells (i.e., eosinophils and goblet cells).

The immune cells infiltration landscape was portrayed using sequencing results of mouse nasal mucosal tissues. Cibersort analysis of mRNA expression showed that compared with the negative control group (PBS group), there were more mast cells in the OVA group. Moreover, compared with OVA group, monocytes, mast cells and neutrophils were decreased in PLGA-Exos group. These results confirmed the successful establishment of study model and the anti-inflammatory effects of PLGA-Exos therapy. Naïve B cells were also increased in the PLGA-Exos group compared to the OVA group, which may imply more bone marrow mobilization (**Figure 4E**; **Supplementary Figure S8A**). In addition, as presented in **Figure 4F** and **Supplementary Figure S8B** when compared with OVA group, Th2 cells were decreased whereas Th1 and Tregs were increased significantly in PLGA-Exos group donating excellent therapeutic effect of exosomes.

### 3.7 PLGA-Exos treatment induces Th1 and Tregs differentiation and depletes Th2 cells in AR mice

**Figure 5:**
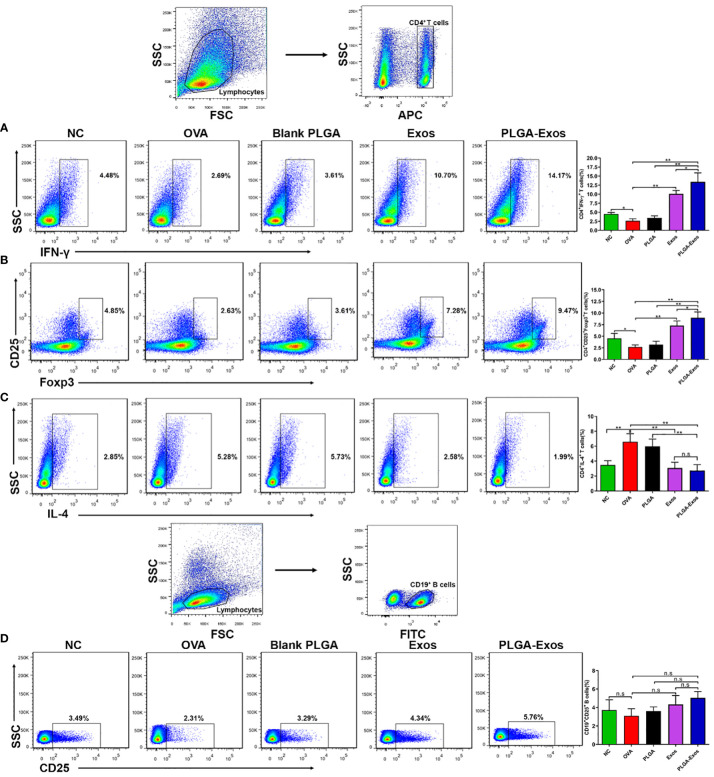
PLGA-Exos therapy at 4 weeks’ time point induces Th1 and Tregs and depletes Th2 cells *in vivo.* **(A)** Frequencies of IFN-γ **(B)** Regulatory T cells **(C)** IL-4 and **(D)** Regulatory B cells in lymphocytes isolated from spleens of AR mice. (NC, negative control/normal mice; OVA, positive control/AR mice; Blank PLGA, AR mice treated with blank PLGA; Exos, AR mice treated with only exosomes; PLGA-Exos, AR mice treated with only exosomes-encapsulated in PLGA sub-micron particles) (*P < 0.05 and ** *P* < 0.01) (n.s, Not Significant).

The cellular changes after PLGA-Exos treatment were assessed by monitoring the counts of Th1/Th2/Tregs/Bregs in the spleen of mice from each treatment group. The results showed increased Th2 and decreased Th1 in OVA-sensitized AR mice. In addition, the percentage of IFN-γ^+^CD4^+^ cells in the spleen were 4.48 ± 0.42, 2.69 ± 0.58, 2.61 ± 0.58, 10.70 ± 0.94 and 14.17 ± 2.45 in PBS, OVA, PLGA, Exos and PLGA-Exos groups, respectively, at 4-weeks (**Figure 5A**). Similarly, Tregs counts were 4.85 ± 1.08, 2.63 ± 0.45, 3.61 ± 0.71, 7.28 ± 0.98 and 9.47 ± 1.25 in PBS, OVA, PLGA, Exos and PLGA-Exos groups, respectively, at 4-weeks (**Figure 5B**). Conversely, the population of IL-4^+^CD4^+^ cells increased to 5.28 ± 1.09 in the OVA group but reduced to 2.58 ± 0.77 and 1.99 ± 0.79 in the Exos alone and PLGA-Exos groups after 6 sessions of treatment (**Figure 5C**). The proportion of Bregs was not statistically different among all the groups at both 2- and 4-weeks (**Figure 5D**; **Supplementary Figure S8D**). However, the proportion of Tregs did increase at 2-weeks’ time point (**Supplementary Figure S9B**). These results revealed that Th1/Th2 disbalance was corrected in Exos alone and PLGA-Exos treated AR mice after 6 doses (4 weeks). In addition, PLGA-Exos treatment exhibited better effect than Exos alone group in terms of reestablishing the Th1/Th2 balance (14.17% > 10.70% for IFN-γ, 1.99% < 2.58% for IL-4) and upregulation of Tregs (9.47% > 7.28% for CD4^+^CD25^+^Foxp3^+^). Being consistent with the flow cytometric results, transcriptomic analysis suggested that the proportion of Th2 cells increased in the OVA group and reduced in the PLGA-Exos group. In addition, the changes of γδT cells, which also secrete IL-17, were similar to the trend of decreased IL-17 as proven by ELISA and RT-qPCR (**Figures 4C, D, F**).

### 3.8 Analysis of exosomal microRNA suggests potential source of the therapeutic effect of PLGA-Exos treatment for AR

**Figure 6:**
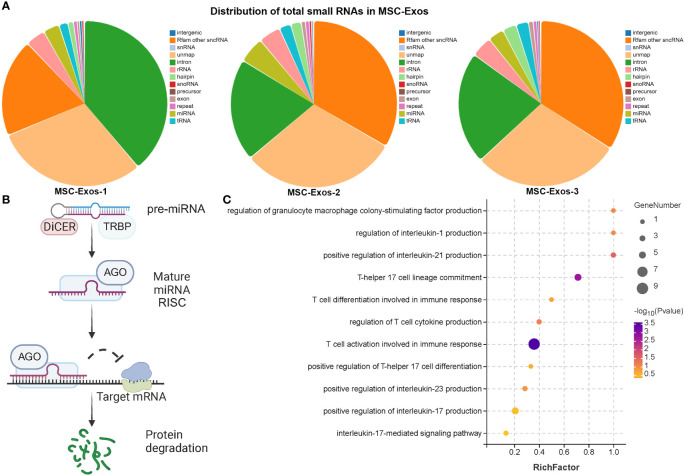
Exosomal microRNA therapeutic effect in AR mice **(A)** The distribution of total small RNAs in MSC-exos **(B)** The diagram of post-transcriptional regulation of microRNA. **(C)** GO analysis of target genes of miRNAs in MSC-Exos. (n=3, performed in triplicate).

Nucleic acids, especially miRNA, are one of the most important functional cargoes of exosomes. As **Figure 6A**; **Supplementary Figure S9A** demonstrated, the proportions of miRNA and length distributions of small RNA were similar among samples. In addition, the sequencing quality of small RNA was acceptable. **Figure 6B** illustrates the mechanism of microRNA involved in post-transcriptional silencing. pre-miRNA, the precursor of microRNA, is about 70-90 bases in length. After Dicer enzyme digestion, pre-miRNA became mature miRNA with a length of 20~24 nt. The mature miRNA binds to the target mRNA and causes its degradation. miRNAs in MSC-Exos might play multiple roles in the treatment of AR, such as inhibition of cytokine production, activation of T cells during immune response, and regulation of GM-CSF production (**Figure 6C**). Interestingly, MSC-Exos might not only inhibit Th17 cell lineage commitment and IL-17 mediated signaling pathway, but also attenuate the differentiation of Th0 to Th17 cells by suppressing cytokines that promote Th17 differentiation, such as IL-1, IL-21 and IL-23 (**Figure 6C**).

### 3.9 Enrichment analysis reveals numerous differentially expressed genes during RNA sequencing of nasal tissues

**Figure 7:**
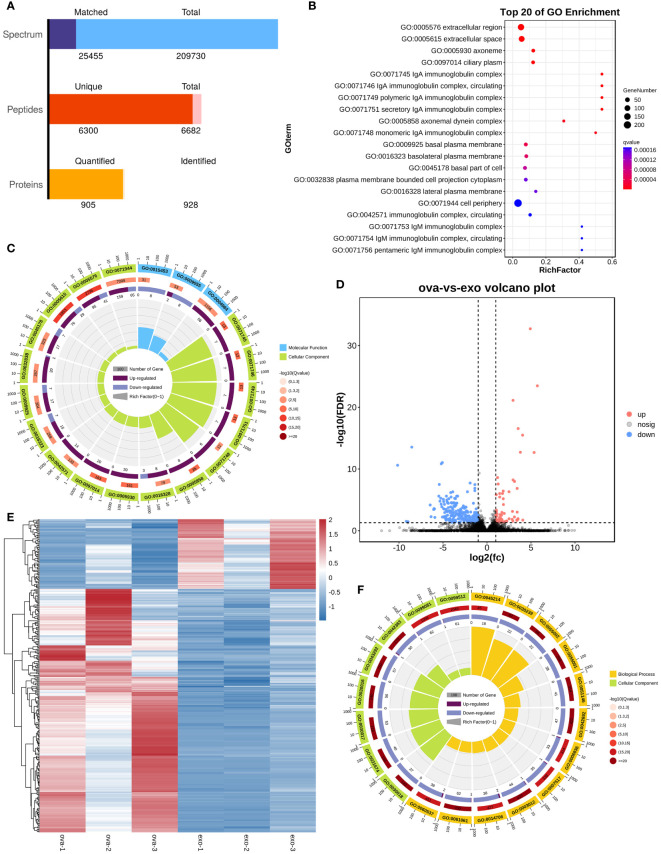
Enrichment analysis of DEG between OVA and Exos groups. **(A)** Peptides identified by untargeted LC–MS analysis in MSC-Exos. **(B)** GO analysis of differentially expressed genes between OVA and NC groups. **(C)** Chord plot of GO analysis between OVA and NC groups. **(D)** Volcano plot of differentially expressed genes between OVA and Exos groups. **(E)** Heatmap of differentially expressed genes between OVA and Exos groups. **(F)** Chord plot of GO analysis between OVA and Exos groups. (n=3).

The boxplots in **Supplementary Figures S10A, B** indicated that the extracted RNA was of good quality and integrity. In addition, the distribution of FPKM of mouse nasal mucosal tissues were similar among different samples (**Supplementary Figures S10C–E**). The protein components of MSC-Exos were detected using non-target mass spectrometry, and a total of 905 quantified proteins were detected (**Figure 7A**). Firstly, our sequencing results validated the successful establishment of an animal model of AR. The bubble plot revealed multiple inflammatory and immune-related pathways in OVA group (e.g., IgA complex, IgM complex, eosinophil activation involved in immune response, and positive regulation of mast cell activation) (**Figure 7B**). The chord plot, showing the enriched pathway in different colors, also showed the total gene number of the pathway and the number of up-regulated and down-regulated genes in detail (**Figure 7C**). Secondly, the differentially expressed genes (DEGs) between the OVA group (AR model) and treatment group (PLGA-Exos) were illustrated by the volcano plots and heatmap (**Figures 7D, E**). Finally, functional enrichment analysis was performed based on the Gene Ontology (GO) annotation (section 3.11) (**Figure 7F**).

### 3.10 Multiomics analysis enriches several signaling pathways underlying the therapeutic effect of PLGA-Exos treatment in AR mice

**Figure 8:**
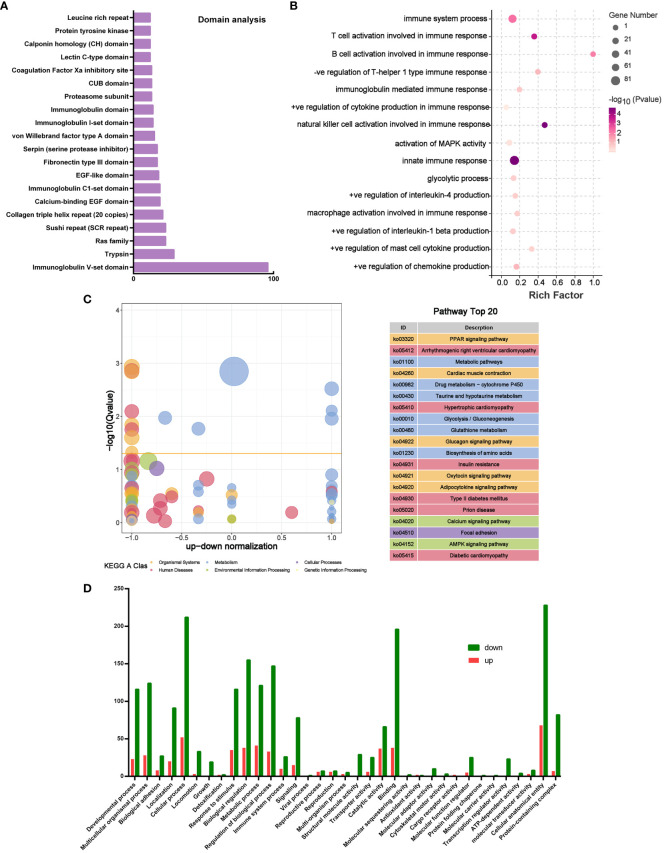
Multiomics analysis of microRNA and mouse nasal mucosal tissues. **(A)** Top 20 Domain analysis of proteins in MSC-Exos. **(B)** Boxplot of GO analysis of differentially expressed genes between OVA and Exos groups. **(C)** KEGG analysis of differentially expressed genes between OVA and Exos groups. **(D)** GO analysis of target genes of miRNAs in MSC-Exos. (n=3).

In order to analyze the potential mechanism of PLGA-Exos treatment of AR, the proteomics data of MSC-Exos, microRNA seq data of MSC-Exos, and transcriptomic data of PLGA-Exos treated mucosal tissue were cross-verified. The domain analysis based on LC-MS showed many immunoglobulin subunits (e.g., Ig V-set domain, Ig C1-set domain and Ig I-set domain) in MSC-Exos (**Figure 8A**). In addition, the miRNA target gene enrichment analysis also revealed multiple inhibited signaling pathways that are related to immune and inflammatory cytokines (e.g., T cell and B cell activation during immune response, activation of Mitogen-activated protein kinase (MAPK) activity, NK cell activation, mast cell cytokine production and innate immune response) (**Figure 8B**). These results supported the anti-inflammatory effect of PLGA-Exos in the treatment of AR. The KEGG analysis of PLGA-Exos treated nasal mucosal tissues demonstrated several potentially crucial signaling pathways (e.g., PPAR pathway, metabolic pathway, glycolysis pathway and AMPK pathway) (**Figure 8C**). In addition, the GO enrichment analysis suggested dozens of important processes such as the metabolism process, immune system process and ATP-dependent activity (**Figure 8D**). Finally, after intersecting three sets of omics data, we identified two important pathways that might explain the profile changes in immune cells and cytokines after PLGA-Exos treatment, i.e., the activation of PPAR signaling pathway and the disruption of glycolysis pathway (**Supplementary Figures S11A, B**).

## 4 Discussion

As recommended by the Allergic Rhinitis and its Impact on Asthma (ARIA) guideline (40, 41), the pharmacotherapy of AR commonly involves oral and intranasal H1-antihistamines, intranasal corticosteroids, and leukotriene receptor antagonists used either alone or in combination. AR is characterized by Th1, Th2 and IgE mediated disorder which involve the activation of mast cells, eosinophils and dendritic cells by hyperresponsiveness of allergens such as pollens, fungal spores, or house-dust mite (42). Conventional medications such as allergen specific immunotherapies or intranasal steroids being used to cure AR are controversial because of their poor compliance with allergen avoidance or their severe side effects (43). Stem cell therapy, especially MSC-based therapy, has demonstrated several advantages, such as multilineage differentiation potential, immunomodulatory effect, migratability to the exact site of injury, and applicability to a wide range of diseases, thereby offering new options for patients suffering from previously incurable disorders (44–47). However, some clinical trials have failed to show any benefit in the practice. This is likely due to the inevitable limitations of stem cell therapy, such as infusion toxicity, immunogenicity, tumorigenic potentials and ethical issues (48).

Recently, exosomes are reported to play a crucial role in cell-to-cell communication and are associated with a wide range of physiological and pathological functions, including modulation of adaptive immune responses (49). Particularly, exosomes derived from different cell sources are documented to contribute in differentiation of naïve CD4^+^ T lymphocytes into Th1 and Th2 cells and cytokines production (50–53). Considering all the aforementioned facts, in present study, exosomes derived from MSCs of human umbilical cord were delivered intranasally to explore their potential anti-inflammatory effect in OVA-induced AR mice.

Intranasal administration of exosomes in case of AR immunotherapy may encounter issues of rapid clearance (54). In drug delivery system, the major concerns for successful delivery are retention time, sustained release and maintained bioactive nature of encapsulated therapeutic drugs (10, 55). Advanced biologic therapeutics, like exosomes, require innovative engineering to deliver (56). PLGA, a biodegradable and biocompatible polymer, is widely used in vaccine and drug delivery systems for humans (27, 35). Previously, PLGA MPs/NPs have been fabricated with varying sizes and morphologies for co-coupling on the surface, as well as for encapsulation of drugs inside the MPs/NPs. This allows controlled and sustained delivery of drugs and other molecules such as RNAs, antibodies, and cytokines to specific organs without causing any organ toxicity issues (26, 57, 58). Recently, a novel platform was developed to encapsulate exosomes in PLGA MPs/SMPs, which enabled a prolonged release of these exosomes to modulate dentiogenesis (55) and periprosthetic osteolysis (10). For the first time, we have developed a controlled and sustained release platform for the treatment of AR by encapsulating exosomes in PLGA MPs/SMPs to minimize the rapid clearance issue of exosomes in nasal mucosa. We hypothesized that this platform is capable of modulating the inflammatory cytokines produced in response to allergens both *in vitro* and in a mouse model. Different sized exosomes encapsulated PLGA MPs/SMPs microspheres were generated by the double emulsion method (**Figure 1**; **Supplementary Figure S2**). First, we evaluated the suitable PLGA MPs/SMPs size with longer retention time and sustained release. Our results demonstrated that PLGA-Exos with 800 nm size and 10 µg/mg concentration were found with longer intranasal retention time and longer sustained release profile for 11 days (**Figure 2**). Based on these findings, in our study, *in vivo* treatment regime consisted of six doses administered at 5-day intervals.

It is reported that exposure to LPS is linked to damage the airway epithelium and elevate the expression of inflammatory cytokines in HNEpCs (59, 60). The *in vitro* therapeutic potential of PLGA-Exos was determined by coincubation of PLGA-Exos with LPS stimulated HNEpCs. These findings revealed that PLGA-Exos significantly inhibited the LPS-induced inflammatory expressions by increasing the anti-inflammatory cytokines (INF-γ, IL-2), decreasing pro-inflammatory cytokines (IL-4) and LPS concentrations (**Figure 3**).

It is established that MSC-Exos treatment showed significant improvement in reducing lung inflammation and airway hyper-responsiveness in a murine model (61). It is commonly assumed that there is a relationship between the levels of mRNA and proteins. However, due to involvement of various complicated post transcriptional changes in the process of translation from mRNA to proteins, this correlation is poor (62). Most cytokines are affected more by compounds on protein levels than mRNA (63). In this study, we found that PLGA-Exos treatment resulted in elevated levels of INF-γ, IL-2, and IL-10, and decreased levels of IL-4, IL-17, and sIgE which indicated similar patterns of cytokine production and mRNA expression (**Supplementary Figures S4A, B**; **Figures 4A, B**). The nasal epithelial tissues showed infiltration of inflammatory cells including eosinophils, basophils, mast cells and goblet cells, which perpetuate the nasal mucosal inflammation in OVA-induced AR mice. Histopathological analysis after intranasal treatment with PLGA-Exos exhibited efficient amelioration in nasal tissue disruption both after 2^nd^ (3 doses) and 4^th^ (6 doses) weeks of treatment (**Supplementary Figures S4C, D**; **Figures 4C, D**).

AR is associated with production of allergen specific-IgE, IL-4 secreted by Th2, delayed Th1 response by downregulation of IL-2 and INF-γ and decreasing the population of CD25^+^Foxp3^+^ regulatory T cells (Tregs) while as the role of regulatory B cells is not clear (64). Exosomes contain the beneficial paracrine effects of stem cells avoiding concerns about immunogenicity (65). Simultaneously, exosomes can play role to regulate inflammatory diseases and cancers by transmission of their nucleic acids, proteins and lipids (66). Flow cytometric characterization was performed to investigate the regulatory mechanism of inflammatory cytokines expression by exosomes treatment more precisely. Our findings confirmed that PLGA-Exos are capable to ameliorate the differentiation of Th1 and Tregs, and inhibit the differentiation of Th2 cells, whereas, Bregs were not induced significantly (**Figure 5**).

We identified two important pathways that might explain the profile changes in immune cells and cytokines after PLGA-Exos treatment, i.e., the activation of PPAR signaling pathway and the disruption of glycolysis pathway (**Figures 8C**). The peroxisome proliferator-activated receptor (PPAR), a member of the nuclear receptor superfamily with three isoforms, i.e., PPARγ, PPARα and PPARδ, is one of the most extensively studied ligand-regulated transcription factors (67). Emerging evidence reveals that PPARs, especially PPARγ, are important for the maturation and function of various immune cells, such as macrophages, DCs, and B cells and T cells. Airway epithelial cells specific PPARγ could regulate airway inflammation and mucin expression in allergic asthma by functioning as a transcriptional repressor of MUC5AC, a major airway mucin gene (68). Additionally, PPARγ agonist could inhibit AR by inducing Tregs, whereas PPARγ antagonists reversed this anti-inflammatory effect (69). Glycolysis is a key player in the pro-inflammatory response since inflammatory cells switch their metabolism towards glycolysis and high lactate production to satisfy their demanding energetic and biosynthetic needs (70). In addition, lactate production is increased in the hypoxic state of CRS and AR, which could in turn enhance the functions of Th cells, macrophages and neutrophils (71). Finally, inhibition of aerobic glycolysis could not only reduce the production of lactate, IL-5, IL-17, and IFN-γ, but also stimulate that of IL-10 and Foxp3 in a mouse model of asthma (72). This is partially because Tregs might utilize collateral pathways such as PPAR pathway, mTOR pathway, AMPK pathway, and other metabolic pathways, as discovered in our multiomics analysis, to reestablish the Th17/Treg balance (73).

## 5 Conclusion and future perspectives

In this preclinical study, we are the first to systematically demonstrate potent immunomodulatory effect of mesenchymal stem cell-derived exosomes delivered using PLGA sub-micron particles for the treatment of allergic rhinitis and their underlying molecular mechanism. Using *in vitro* models (RPMI2650 and THP-1 cells) and *in vivo* model (Balb/c mice), we first established that encapsulating MSC-Exos in PLGA improved retention time and achieved sustained local release for intranasal administration. Further phenotypic and mechanistic studies revealed that PLGA-Exos could provide significant immunomodulatory effects in LPS-stimulated HNEpCs cellular model and OVA-provoked murine model of AR. This was manifested hierarchically: dampened infiltration of immune cells (e.g., eosinophils and goblet cells) in the nasal mucosa at tissue level, increased differentiation of Tregs and Th1 cells, reduced Th2 and Th17 cells, and corrected Th1/Th2 disbalance at cellular level, and suppressed pro-inflammatory cytokines and promoted anti-inflammatory cytokines at protein level. Assisted by bioinformatic analysis, we discovered numerous signaling pathways that are sensitive to PLGA-Exos treatment of AR, such as IL-17/Th17 pathway, PPAR pathway, and glycometabolism and glycolysis pathway, most of which have interesting and extensive connection and interaction. Most importantly, PLGA-Exos inherit similar therapeutic effects from their parental MSCs without exhibiting the disadvantages of these stem cells. In conclusion, MSC-Exos locally delivered using our PLGA platform is a promising, adjunctive, immunomodulatory AR therapy. Based on the transcriptomic, protein, cellular and animal data from this study, the ideal next stage would be preparation for human clinical trials. The prospects for the future treatment of allergic rhinitis with exosomes are as follows. Firstly, we need to determine whether exosomes are safe for clinical trials. Secondly, whether the MSCs donor for exosomes isolation may carry any genetic or infectious disease. In addition, extensive research is needed to approve exosomes as therapeutic agent in human body and their best possible route of administration depending upon the nature of disease.

## Data availability statement

The datasets presented in this study can be found in online repositories. The names of the repository/repositories and accession number(s) can be found below: https://www.ncbi.nlm.nih.gov/, PRJNA1032242 http://www.proteomexchange.org/, PXD046568.

## Ethics statement

Ethical approval was not required for the studies on humans in accordance with the local legislation and institutional requirements because only commercially available established cell lines were used. The animal study was approved by the School of Medicine, Tongji University (Permit no. T3-HB-LAL-2023-23) and recommendations of Guide for the Care and Use of Laboratory Animals of Ministry of Science and Technology of the People’s Republic of China were followed. The study was conducted in accordance with the local legislation and institutional requirements.

## Author contributions

KS: Conceptualization, Formal analysis, Methodology, Writing – original draft. ZW: Conceptualization, Funding acquisition, Methodology, Writing – review & editing. XL: Methodology, Software, Writing – review & editing. JL: Methodology, Writing – review & editing. MX: Software, Writing – original draft. FT: Conceptualization, Funding acquisition, Supervision, Writing – review & editing.
