# Supplementary material for: Immunomodulatory effect of PLGA-encapsulated mesenchymal stem cells-derived exosomes for the treatment of allergic rhinitis
Source: Front Immunol. 2024 Jul 8;15:1429442. doi: 10.3389/fimmu.2024.1429442 (PMC11260627; doi:10.3389/fimmu.2024.1429442)
Supplement: Supplementary file 1 [file DataSheet_1.docx]

**Supplementary Data File**

**Immunomodulatory effect of PLGA-encapsulated mesenchymal stem cells-derived exosomes for the treatment of allergic rhinitis**

**Table S1:** Primer sequences for HNEpCs RT-qPCR

| **Name** | **Froward primer (5’-3’)** | **Reverse primer (5’-3’)** |
| --- | --- | --- |
| IL-10 | TCAAGGCGCATGTGAACTCC | GATGTCAAACTCACTCATGGCT |
| IL-2 | TACAAGAACCCGAAACTGACTCG | ACATGAAGGTAGTCTCACTGCC |
| IFN-γ | TGAATGTCCAACGCAAAGCA | CTGGGATGCTCTTCGACCTC |
| IL-4 | AACGACCCGGCAGATTTCAG | GGCTCCCTGTAGGAGTTGTG |
| GAPDH | TGAGGTCAATGAAGGGGTCGT | CCTCGTCCCGTAGACAAAATG |

**Table S2:** Primer sequences for mice RT-qPCR

| **Name** | **Froward primer (5’-3’)** | **Reverse primer(5’-3’)** |
| --- | --- | --- |
| IL-10 | CGCCAAGGGAGTTAAAGACTT | AGGGGAGAAATCGATGACAG |
| IL-2 | AACCTGAAACTCCCCAGGAT | TCATCGAATTGGCACTCAAA |
| IFN-γ | ATGAACGCTACACACTGCATC | CCATCCTTTTGCCAGTTCCTC |
| IL-17 | TCAGCGTGTCCAAACACTGAG | CGCCAAGGGAGTTAAAGACTT |
| IL-4 | GGTCTCAACCCCCAGCTAGT | GCCGATGATCTCTCTCAAGTGAT |
| GAPDH | CATCACTGCCACCCAGAAGACTG | ATGCCAGTGAGCTTCCCGTTCAG |


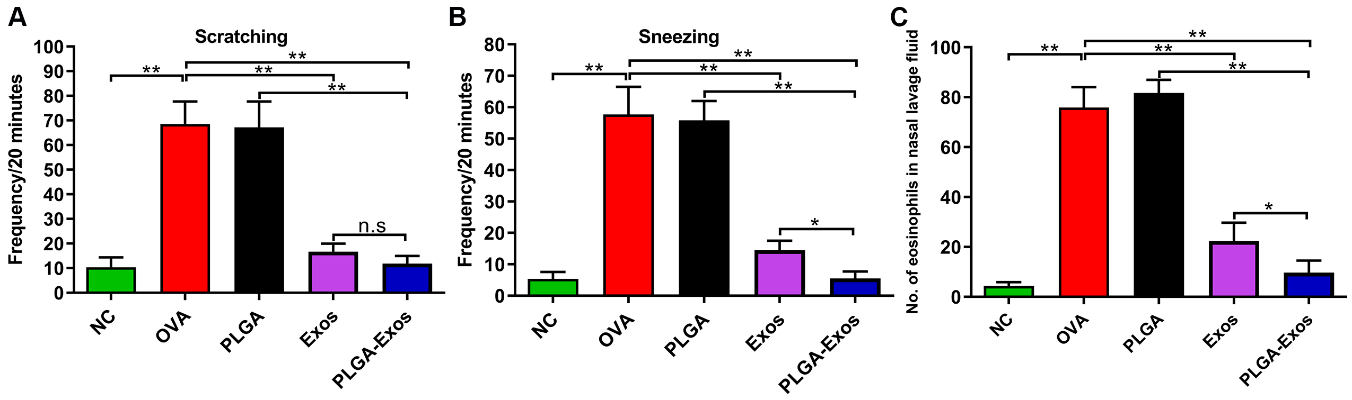


**Figure S1.** AR symptoms. (A) Frequency of scratching and (B) sneezing in different treatment groups for 20 minutes after the last treatment. (C) Number of eosinophils in nasal lavage fluid. (NC= negative control/normal mice; OVA= positive control/AR mice; Blank PLGA= AR mice treated with blank PLGA; Exos= AR mice treated with only exosomes; PLGA-Exos= AR mice treated with only exosomes-encapsulated in PLGA sub-micron particles) (**P* < 0.05 and ** *P* < 0.01).


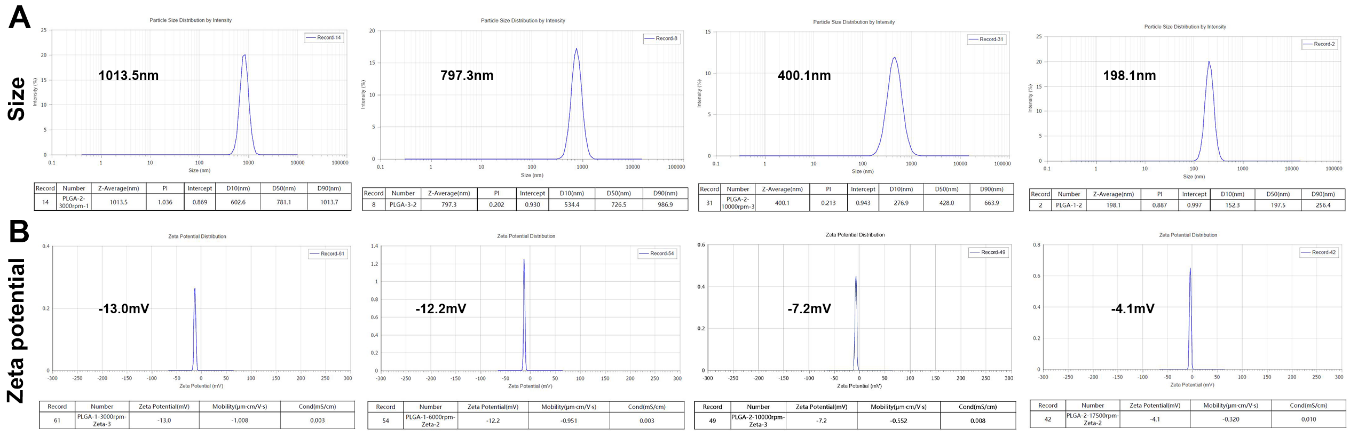


**Figure S2.** Characterization and phenotypic analysis of different-sized blank PLGA MPs/SMPs. (A) Size distribution and (B) zeta potential analysis of different-sized blank PLGA MPs/SMPs by DLS. Each experiment was repeated in triplicate.


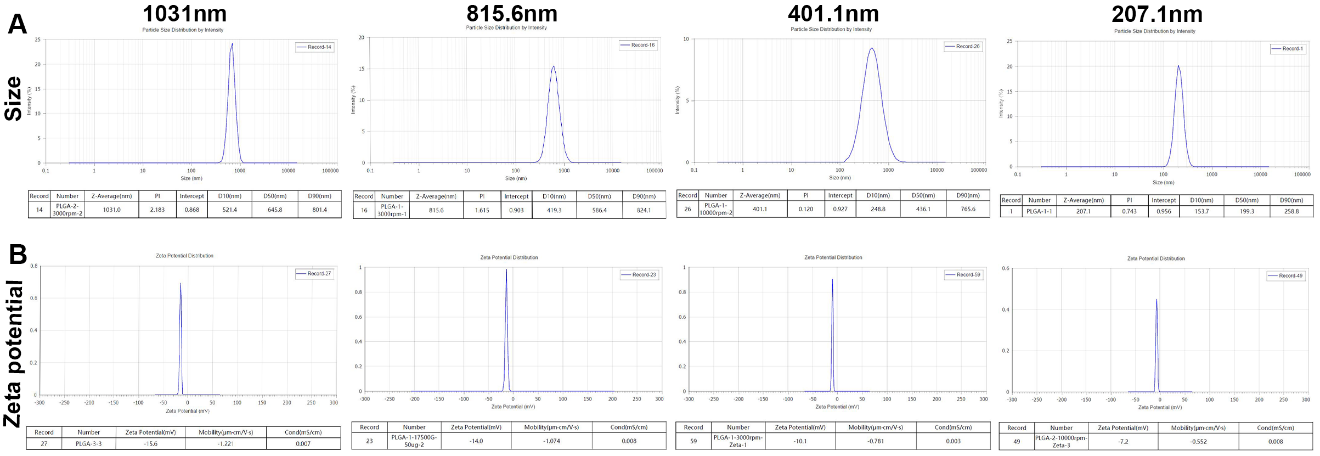


**Figure S3.** Characterization and phenotypic analysis of ICG-encapsulated different-sized PLGA MPs/SMPs. (A) Size distribution and (B) zeta potential analysis of ICG-encapsulated different-sized PLGA MPs/SMPs by DLS. Each experiment was repeated in triplicate.


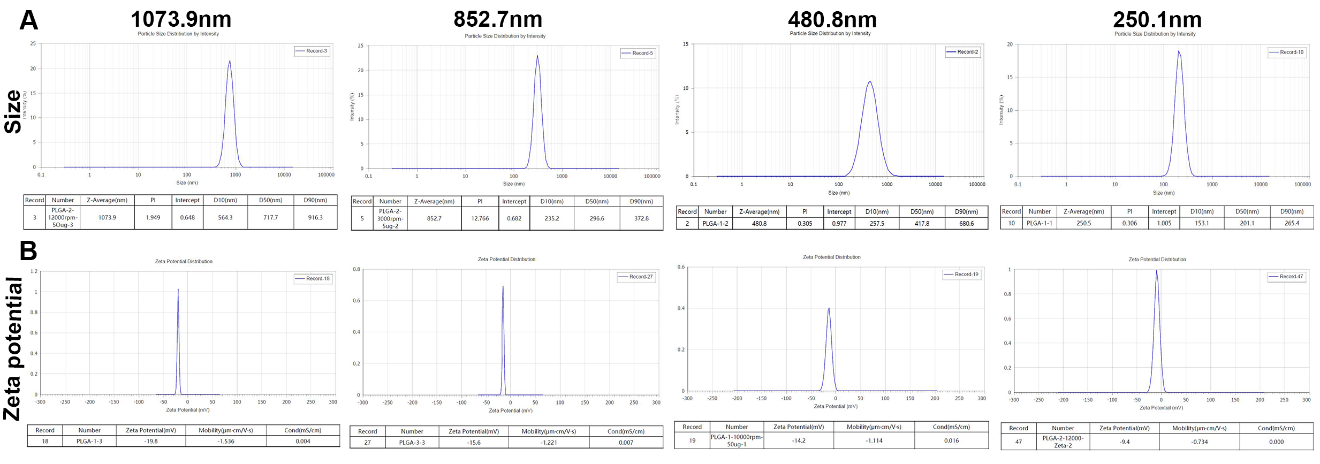


**Figure S4.** Characterization and phenotypic analysis of exosomes encapsulated different-sized PLGA MPs/SMPs (PLGA-Exos). (A) Size distribution and (B) zeta potential analysis of different-sized PLGA-Exos MPs/SMPs by DLS. The representative figures are given for 10 µg/mg concentration of exosomes encapsulated in different-sized PLGA MPs/SMPs. Each experiment was repeated in triplicate.


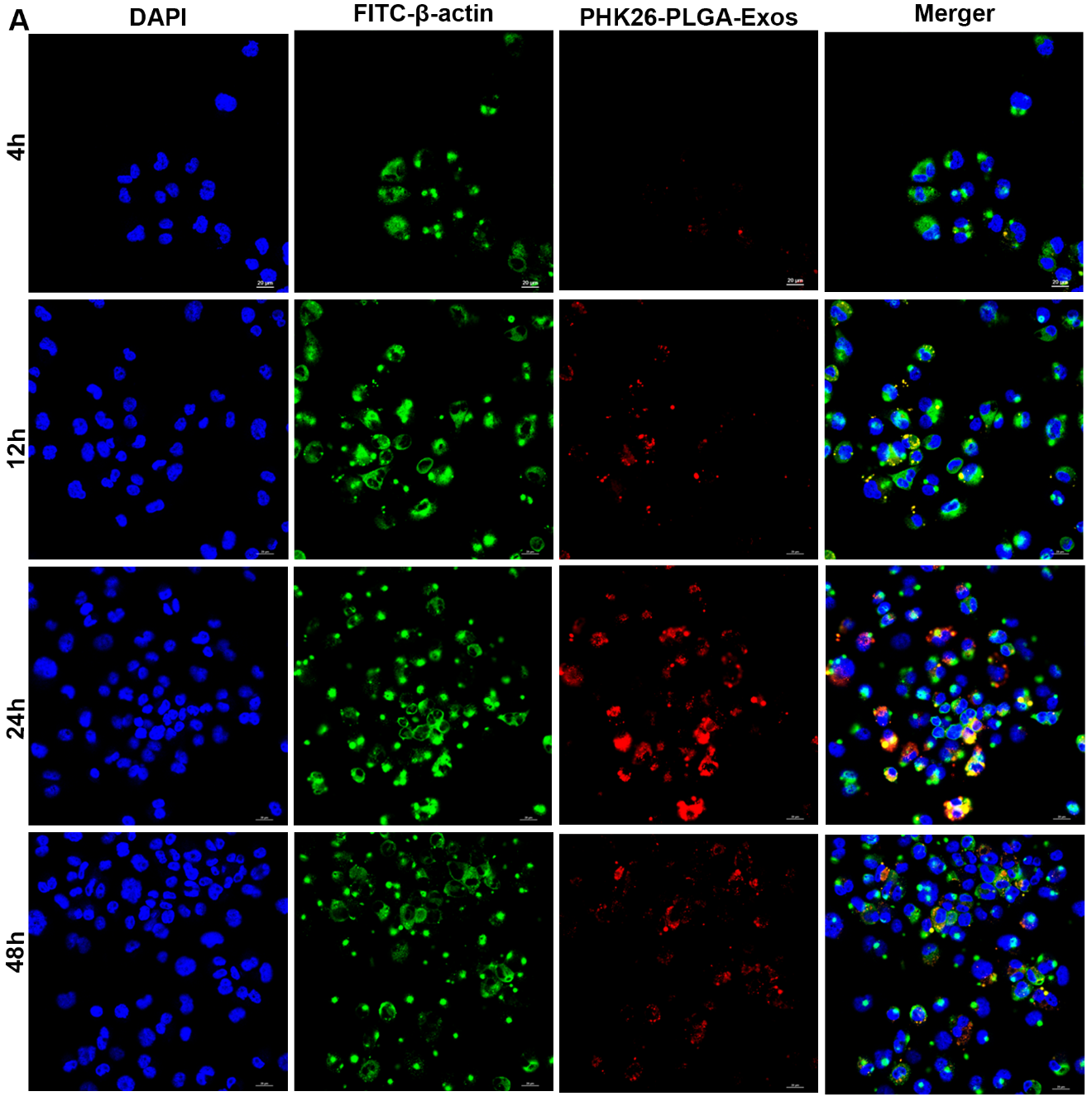


**Figure S5.** Cellular uptake of PLGA-Exos. (A) Immunofluorescence images by confocal microscopy presenting the internalization of PLGA-Exos in RPMI2650 cells; scale bar 20µm. (n=3, performed in triplicate).


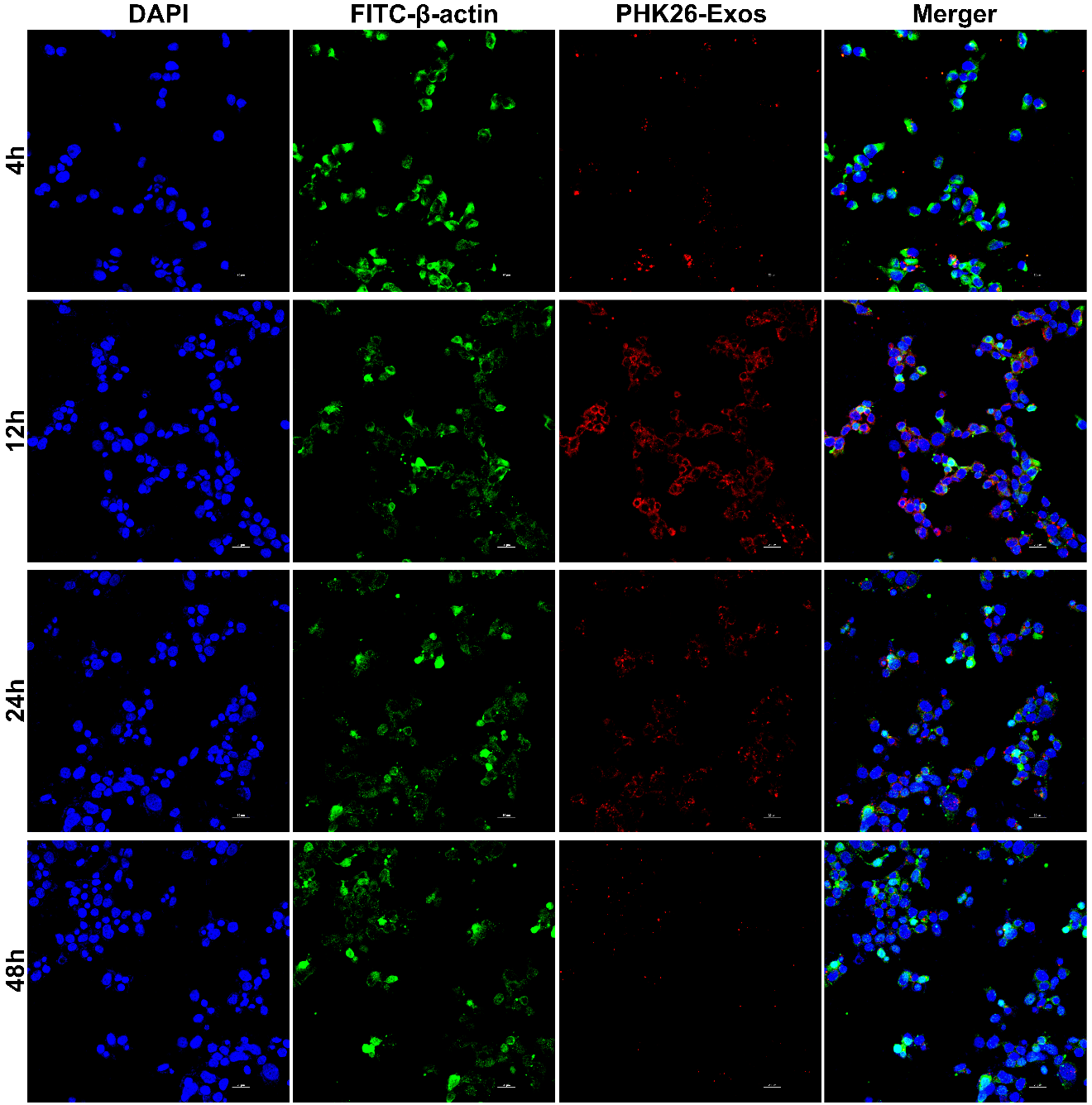


**Figure S6.** Cellular uptake of exosomes. (A) Immunofluorescence images by confocal microscopy presenting the internalization of exosomes in THP-1 cells; scale bar 20µm. (n=3, performed in triplicate).


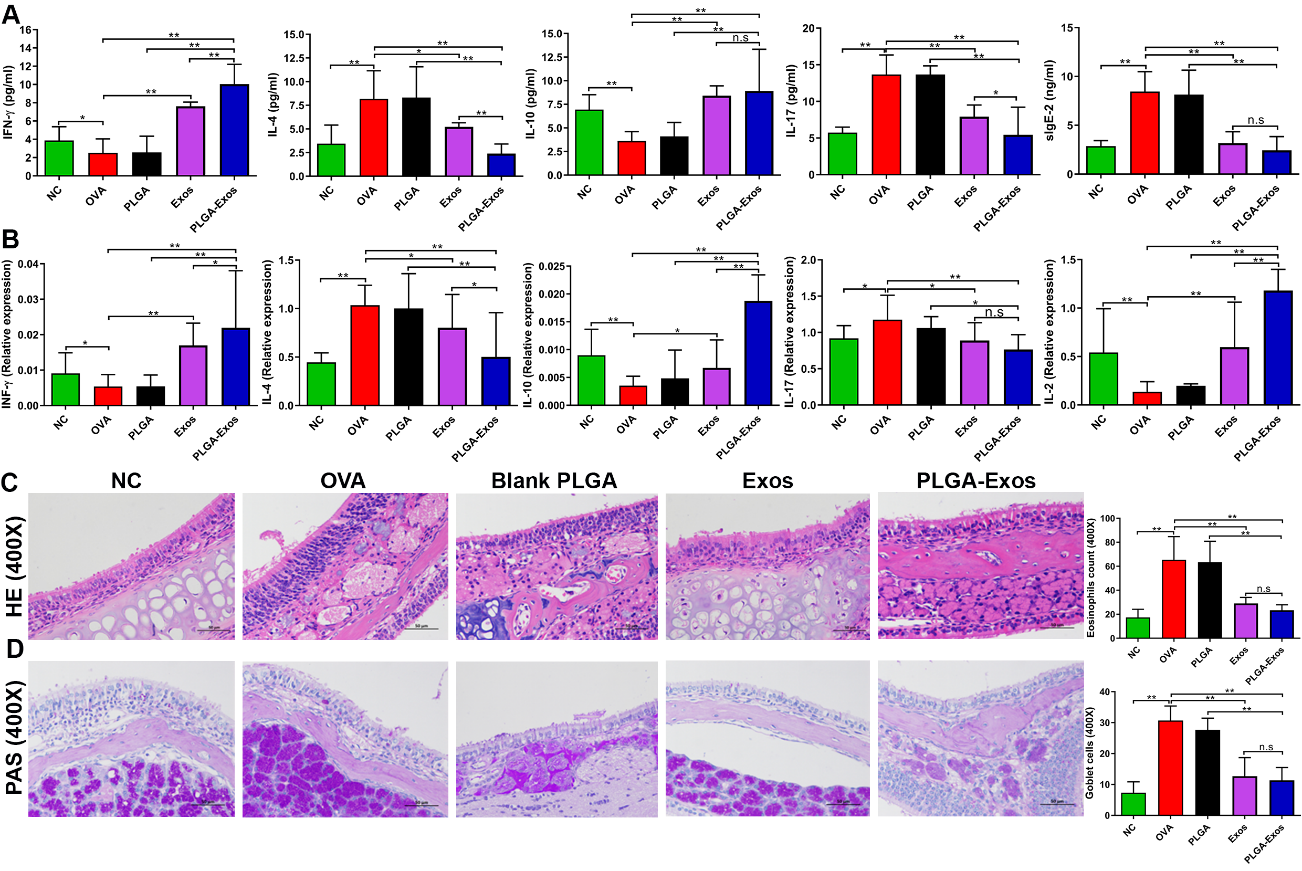


**Figure S7.** Effect of PLGA-Exos treatment on histopathological changes in nasal tissues, blood serum, and spleen cells in AR mice at 2 weeks’ time point. (A) Quantification of INF-γ, IL-4, IL-10, IL-17, and sIgE in blood serum of AR mice by ELISA. (B) Relative mRNA expression levels for INF-γ, IL-4, IL-10, IL-17, and IL-2 in spleen cells of AR mice. (C, D) Representative images of HE and PAS staining of nasal tissues representing the infiltrations of inflammatory cells (eosinophils and goblet cells) in OVA group, which were reduced in treatment groups (Exos and PLGA-exos); Magnification 400X. (NC= negative control/normal mice; OVA= positive control/AR mice; Blank PLGA= AR mice treated with blank PLGA; Exos= AR mice treated with only exosomes; PLGA-Exos= AR mice treated with only exosomes-encapsulated in PLGA sub-micron particles) (n=6) (**P* < 0.05 and ** *P* < 0.01).


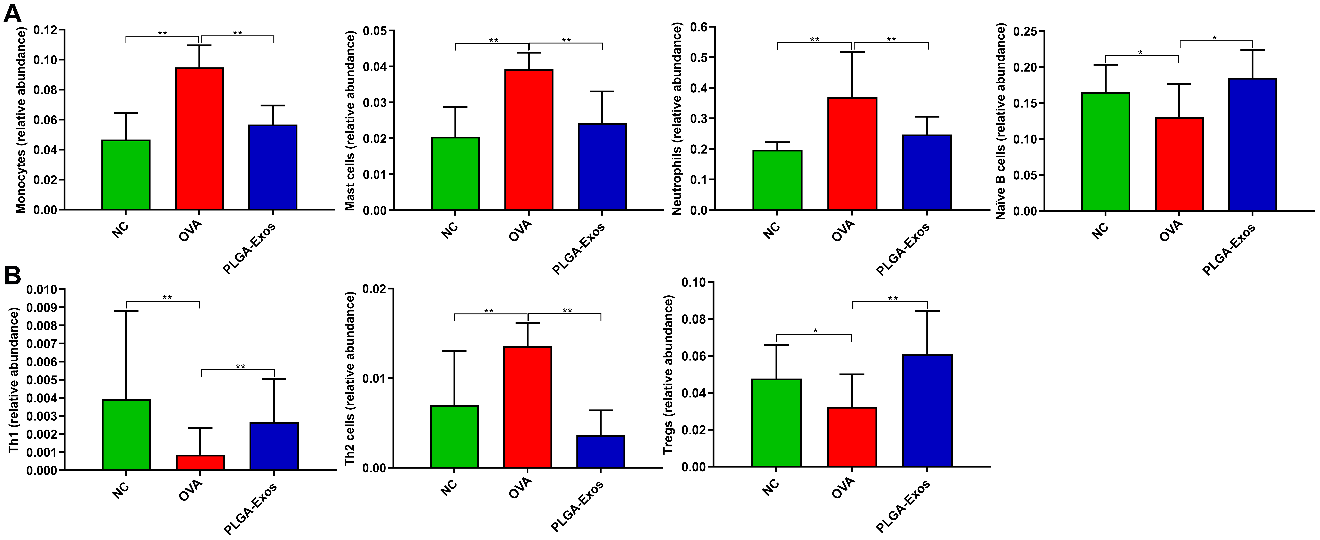


**Figure S8.** Sequencing analysis of immune cells infiltration in mouse nasal mucosal tissues after PLGA-Exos therapy at 4 weeks’ time point*.* (A) Cibersort analysis of the relative abundance of myeloid cells. (B) Cibersort analysis of the relative abundance of lymphoid cells. (NC= negative control/normal mice; OVA= positive control/AR mice; PLGA-Exos= AR mice treated with only exosomes-encapsulated in PLGA sub-micron particles) (n=3) (**P* < 0.05 and ** *P* < 0.01).


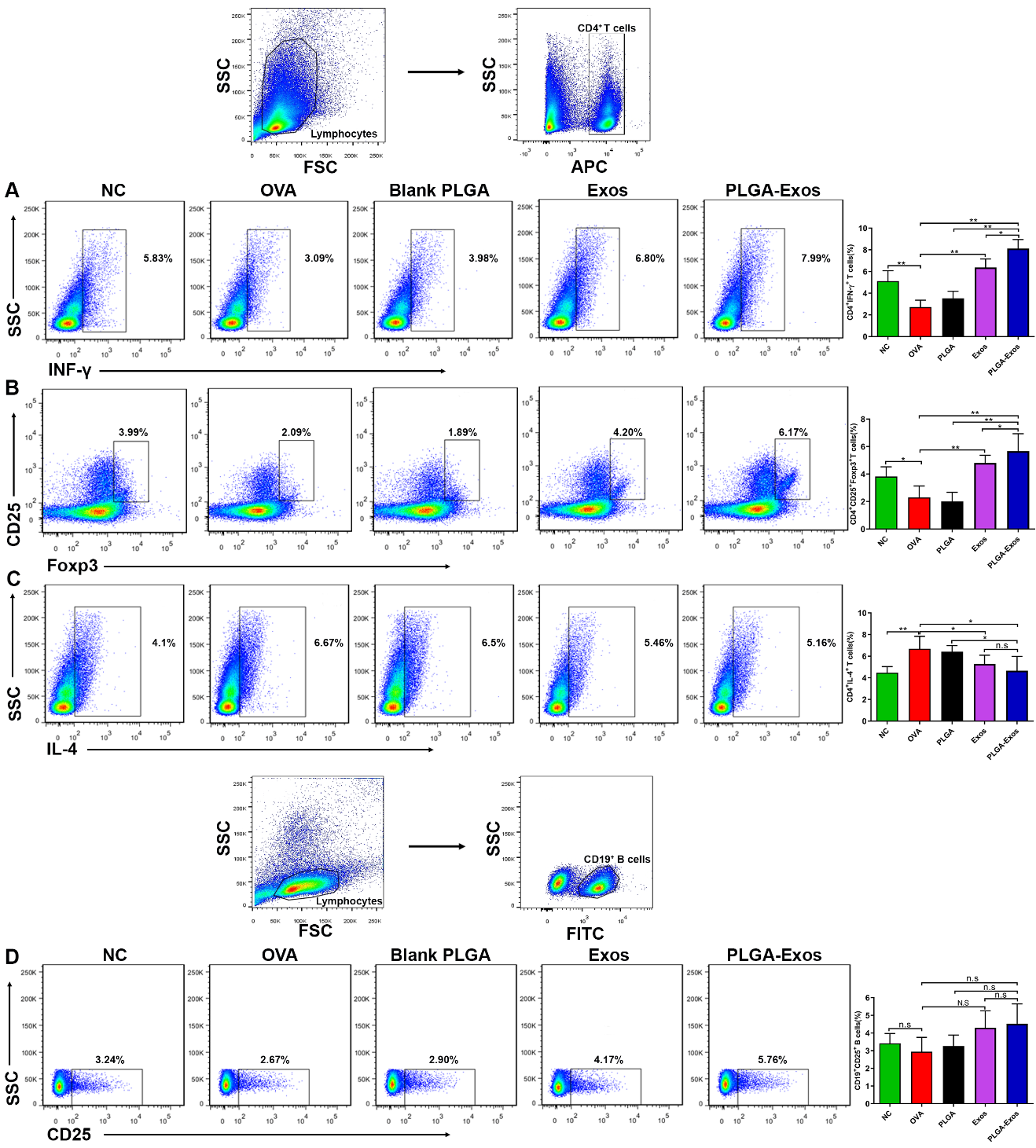


**Figure S9.** PLGA-Exos therapy at 2 weeks’ time point induces Th1 and Tregs and depletes Th2 cells *in vivo.* (A) Frequencies of INF-γ (B) Regulatory T cells (C) IL-4 and (D) Regulatory B cells in lymphocytes isolated from spleens of AR mice. (NC= negative control/normal mice; OVA= positive control/AR mice; Blank PLGA= AR mice treated with blank PLGA; Exos= AR mice treated with only exosomes; PLGA-Exos= AR mice treated with only exosomes-encapsulated in PLGA sub-micron particles) (**P* < 0.05 and ** *P* < 0.01).


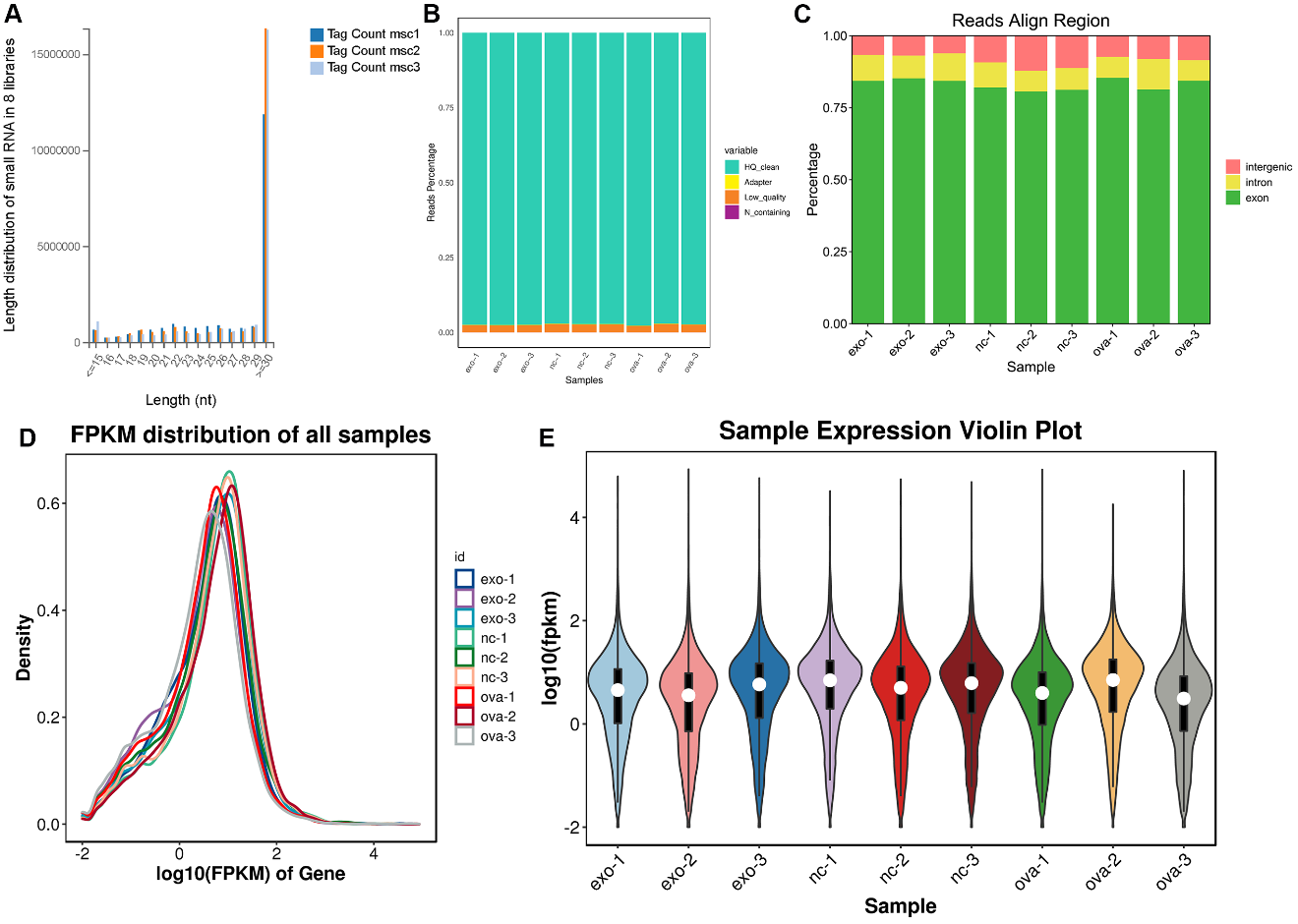


**Figure S10.** The quality assurance steps of RNA sequencing in MSC-Exos and in nasal tissues after treatment with MSC-Exos. (A) Length distribution of small RNA in MSC-Exos. (B) Reads percentage of all samples. (C) The proportion of exon, intron, and intergenic among all samples. (D) The FPKM distribution of all samples. (E) Violin plot of the expression level of all samples. (NC= negative control/normal mice; OVA= positive control/AR mice; Exos= AR mice treated with only exosomes-encapsulated in PLGA sub-micron particles) (n=3)


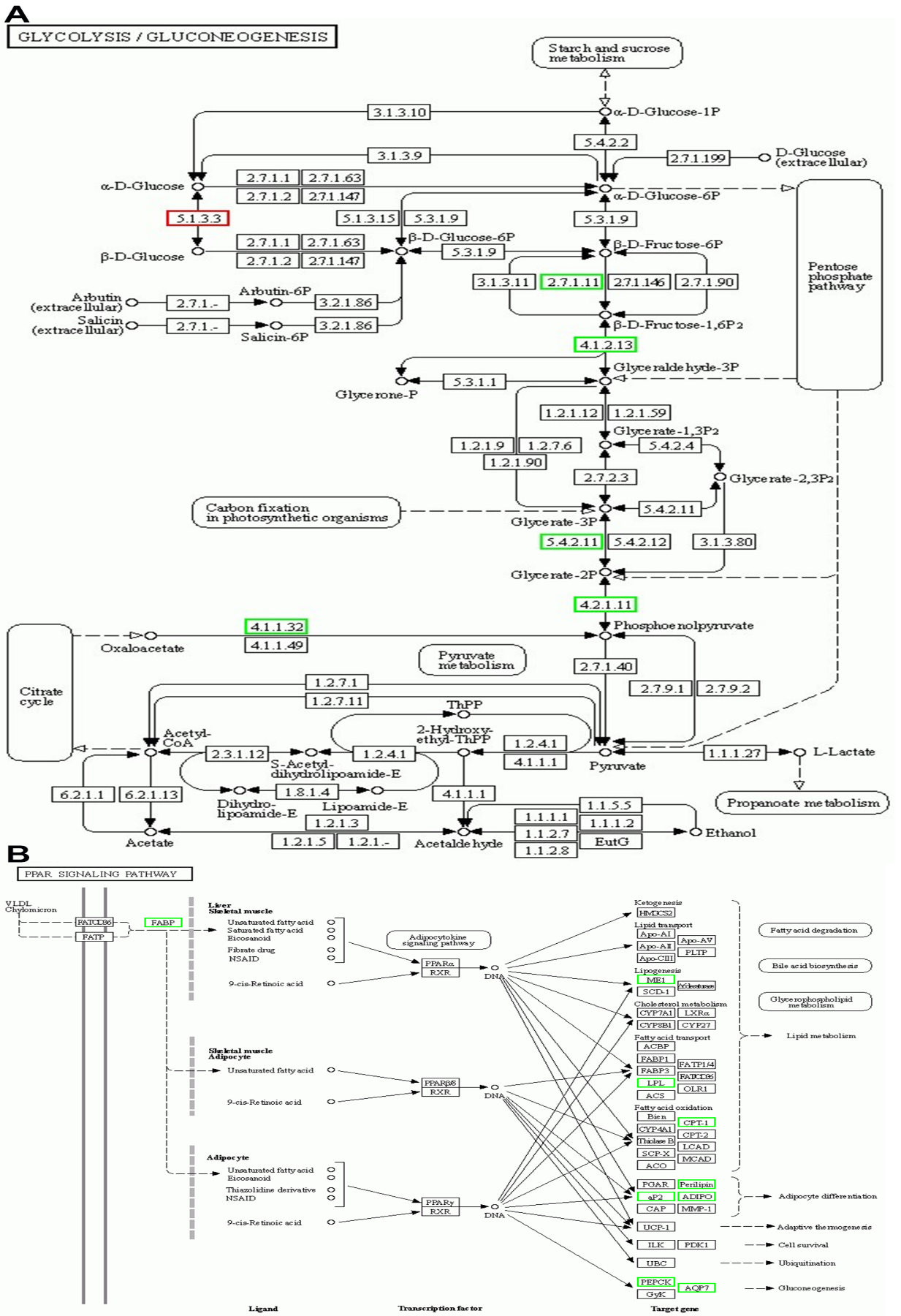


**Figure S11.** (A) KEGG of PPAR signal pathway (B) KEGG of glycolysis pathway. The red color represents up-regulated expression, the green color represents down-regulated expression while white/no color represents no change. (n=3)


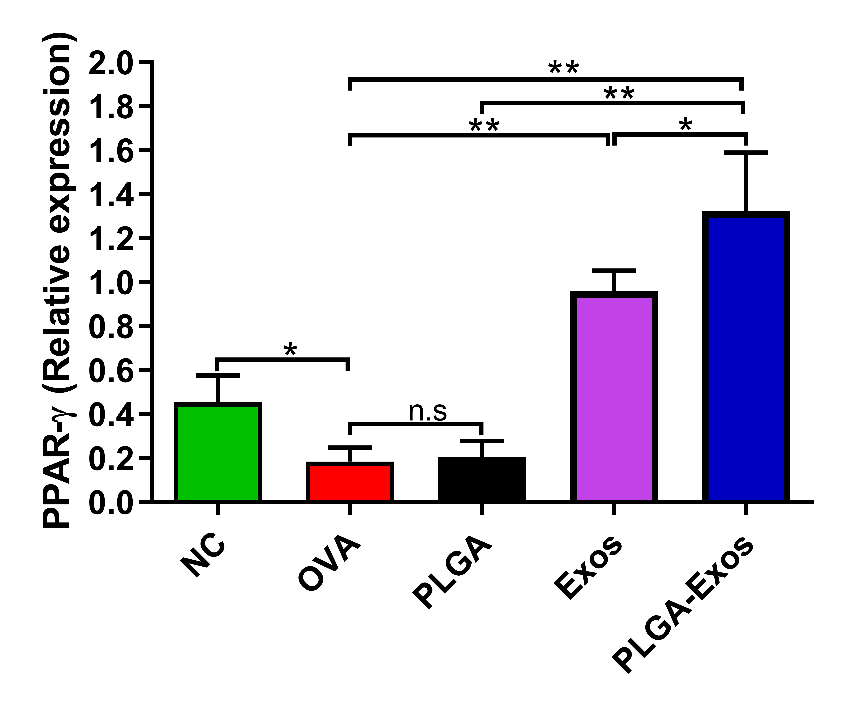


**Figure S12.** Effect of PLGA-exos treatments on spleen cells in AR mice at 4 weeks’ time point. Relative mRNA expression levels for PPAR-γ in spleen cells of AR mice. (NC= negative control/normal mice; OVA= positive control/AR mice; Blank PLGA= AR mice treated with blank PLGA; Exos= AR mice treated with only exosomes; PLGA-Exos= AR mice treated with only exosomes-encapsulated in PLGA sub-micron particles) (n=6) (**P* < 0.05 and ** *P* < 0.01).
